# Health literacy of primary caregivers of children with cerebral palsy in low- and middle-income countries: a systematic review

**DOI:** 10.1136/bmjopen-2024-091679

**Published:** 2025-03-03

**Authors:** Genevieve Lilian Perrins, Catherine King, Kousar Azhdari, Israt Jahan, Rosalie Power, Rashidul Hashan, Nadia Badawi, Gulam Khandaker

**Affiliations:** 1 Central Queensland Public Health Unit, Central Queensland Public Health Unit, Rockhampton, Queensland, Australia; 2 The University of Sydney Faculty of Medicine and Health, Sydney, New South Wales, Australia; 3 The University of Sydney School of Public Health, Sydney, New South Wales, Australia; 4 Faculty of Health, Queensland University of Technology, Brisbane, Queensland, Australia; 5 Central Queensland Public Health Unit, Rockhampton, Queensland, Australia; 6 Medical and Applied Sciences, Central Queensland University, Rockhampton, Queensland, Australia; 7 Translational Health Research Institute, University of Western Sydney, Penrith, New South Wales, Australia; 8 School of Health, Medical and Applied Sciences, Central Queensland University, Rockhampton, Queensland, Australia; 9 Grace Centre for Newborn Intensive Care, Children’s Hosital at Westmead, Sydney, New South Wales, Australia; 10 Cerebral Palsy Alliance, Forestville, New South Wales, Australia; 11 Faculty of Medicine and Health, The University of Sydney, Sydney, New South Wales, Australia

**Keywords:** Caregivers, Child, Health Literacy, Developmental neurology & neurodisability

## Abstract

**Abstract:**

**Objective:**

We aimed to synthesise existing literature on the health literacy of primary caregivers (PCGs) of children with cerebral palsy (CP) in low- and middle-income countries (LMICs).

**Design:**

Systematic review informed by Preferred Reporting Items for Systematic Reviews and Meta-Analyses guidelines.

**Data sources:**

Ovid MEDLINE, Ovid EMBASE, CINAHL via EBSCO, Scopus and Web of Science were searched from inception to January 2024.

**Eligibility criteria:**

Original studies including observational or experimental data, examining health literacy and/or health literacy proxies using Optimising Health Literacy and Access domains as indicators (eg, education, social support, self-efficacy, health attitudes, reading and writing skills) in primary caregivers of children with CP in LMICs.

**Data extraction and synthesis:**

Data from included studies were systematically recorded using an Excel template, with information extracted independently by two reviewers. We used the Study Quality Assessment Tool developed by the National Health, Lung, and Blood Institute.

**Results:**

The systematic review yielded 2734 articles, with 15 eligible for inclusion. None used health literacy (HL) measurement tools, and there was limited reporting on specific HL domains. Studies spanned 11 LMICs across 5 major regions. PCGs, predominantly mothers, exhibited varying levels of service awareness, service utilisation and social support. Literacy levels significantly impacted HL proficiency, exposing a notable research gap in LMICs.

**Conclusions:**

This study presents the first comprehensive analysis of health literacy among PCGs of children with CP in LMICs. Findings reveal a striking absence of tailored health literacy literature, impacting current considerations of PCGs’ understanding and management of their child’s condition. Additionally, challenges in social support, healthcare navigation and low literacy levels further hinder effective caregiving in LMICs.

**STRENGTHS AND LIMITATIONS OF THIS STUDY**

- This review has used a novel approach to filling a knowledge gap relating to health literacy of primary caregivers of children with cerebral palsy in low- and middle-income countries.
- We used the nine Optimising Health Literacy and Access domains of health literacy as proxies for health literacy, which allowed us to broaden the scope of included concepts to provide a comprehensive view of health literacy for this cohort.
- Proxies can introduce imprecision in measured concept and discrepancies through researcher interpretation.
- Due to the absence of health literacy measurements, data were vastly heterogenous and therefore a meta-analysis could not be undertaken.

## Introduction

 Cerebral palsy (CP) is a group of disorders resulting from neurological injury or disruption during the prenatal or early infancy stages of development.^1^ It is a permanent, non-progressive condition that leads to limitations in posture and movement. CP is recognised as the leading cause of childhood physical disability worldwide, with a disproportionately higher prevalence in low- and middle-income countries (LMICs).^2^ In LMICs, an estimated 90% of children with neurodevelopmental disorders, like CP, face service gaps and are primarily managed by their caregivers at home.^3 4^ The health literacy of those primary caregivers significantly influences the quality of care provided to their children. However, there is limited literature about the HL of primary caregivers of children with CP in LMICs.

The WHO defines health literacy as the ‘cognitive and social skills which determine the motivation and ability of individuals to gain access to, understand and use information in ways which promote and maintain good health’,^5^ Health literacy is considered a key factor in determining health outcomes, particularly in complex or compromised health situations such as those involving CP, where it has the potential to empower individuals and their caregivers to effectively manage the condition and enhance overall quality of life.^6^

In situations where an individual is unable to make their own health decisions due to social pressures, deference to health professionals, age or cognitive limitations, the health literacy of a primary caregiver plays a crucial role in overall health and well-being.^7 8^ Research has demonstrated that the health literacy of parents can significantly impact the management of chronic conditions in children, affecting disease progression and quality of life^9^ as well as access to rehabilitation services for children with disabilities.^10^ Children of caregivers with low health literacy are more likely to experience poorer health outcomes and engage less in protective behaviours, underscoring the significance of caregiver health literacy in managing lifelong conditions and promoting the health and well-being of children with CP.^9^

Health literacy is perhaps even more critical for families of children with CP in LMICs as there may be limited understanding of what CP is, risk factors, symptoms, comorbidities and potential management options.^11^ Furthermore, in LMICs, healthcare systems could be complex, expensive and resource-constrained,^12^ and having health literacy gives primary caregivers (PCGs) the tools to make informed decisions about their child’s care. Being health literate helps parents avoid harmful practices, ignore unhelpful advice and discern which services and management strategies are effective and appropriate. They can also advocate for their child and collaborate with health professionals on planning their child’s management. Given the right information, parents can recognise the value of early detection and early intervention.

Over the past few years, some studies and reviews have been conducted to understand the health literacy of primary caregivers and its role in child health-related outcomes.^13^,^15^ Previous reviews have examined the health literacy of parents of children with ‘special needs’,^14^ children with chronic illness^15^ and children with developmental disability.^13^ However, all these studies are high-income country-focused; none are inclusive of parents of children with CP in LMICs. We aimed to consolidate and synthesise existing literature on the health literacy of primary caregivers of children with CP in LMICs. Additionally, we evaluated available literature, assessed methodological strengths and weaknesses and identified potential areas for further academic exploration.

## Methods

**Figure 1:**
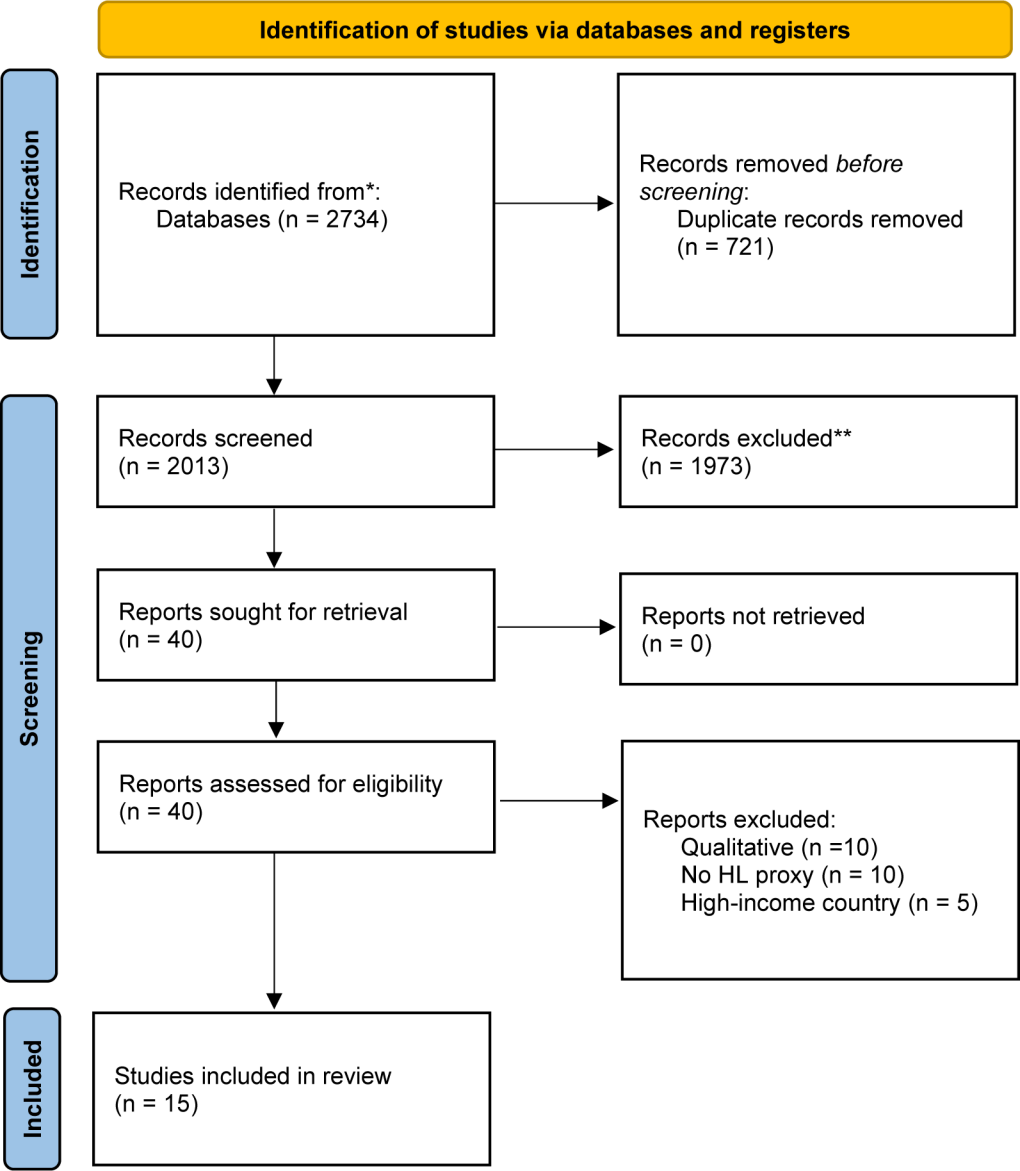
Preferred Reporting Items for Systematic Reviews and Meta-Analyses flow chart. *Consider, if feasible to do so, reporting the number of records identified from each database or register searched (rather than the total number across all databases/registers). **If automation tools were used, indicate how many records were excluded by a human and how many were excluded by automation tools. From: Page *et al*.^37^

We conducted a systematic review with thematic synthesis of data to generate consolidated evidence on health literacy among primary caregivers of children with CP in LMICs. We followed Preferred Reporting Items for Systematic Reviews and Meta-Analyses reporting guidelines (figure 1) and the study protocol was prospectively registered with PROSPERO (protocol number: 42023455560). As a systematic review, there was no patient and public involvement in research design nor data collection.

An initial scoping search revealed a dearth of literature pertaining exclusively to health literacy of PCGs in LMICs. The authors opted to use the multidimensional framework of the Health Literacy Questionnaire (HLQ)—used as part of the ‘Optimising Health Literacy and Access’ (Ophelia) process—developed by Osborne *et al* to categorise available literature.^16^ The Ophelia process was designed to facilitate system redress of health literacy deficits in distinct communities. As a standardised process, its aim is to identify needs of the community, and—adhering to codesign principles—create meaningful change within health organisations to address these needs. With this objective in mind, use of the HLQ provides a holistic and pragmatic health literacy profile, giving users the ability to accurately conceptualise strengths and weaknesses across nine distinct facets of health literacy.

The use of the HLQ/Ophelia domains was not designed for systematic review; however, given the heterogeneity of the literature, a framework was necessary to synthesise and comprehend available information. Given the precision of the HLQ, we opted to use these domains as proxy benchmarks with which to assess available literature. As a process, Ophelia is concerned with measuring health literacy in a way that it can be addressed; it avoids the highly theoretical, abstract notions, instead preferencing a practical approach to measuring health literacy. Furthermore, the Ophelia process and HLQ are exemplified by the WHO for use in LMICs to evaluate and address health literacy in these settings.^17^ For these reasons, Ophelia was selected over other available tools.

### Search strategy

We searched six bibliographic databases in collaboration with an information scientist (CK). The databases included Ovid MEDLINE (1946–18 January 2024), Ovid EMBASE (1947–18 January 2024), CINAHL via EBSCO (1982–20 January 2024), Scopus (1788–20 January 2024) and Web of Science Core Collection (Science Citation Index Expanded (1900–20 January 2024), Social Sciences Citation Index (1956–20 January 2024), Arts and Humanities Citation Index (1975–20 January 2024), Conference Proceedings Citation Index—Science (1990–20 January 2024), Conference Proceedings Citation Index—Social Science and Humanities (1990–20 January 2024), Emerging Sources Citation Index (2005–20 January 2024), Current Chemical Reactions (1985–20 January 2024), Index Chemicus (1993–20 January 2024)). There were no restrictions on publication dates or languages.

Where available, we used controlled vocabulary terms alongside text words such as health literacy, self-efficacy, attitudes and beliefs in combination with mother, father, grandmother, grandfather and children with CP. The full search strategy is available in online supplemental table 1.

#### Selection of articles

Studies were included in this review if they were human studies; original research; observational or experimental studies; involving primary caregivers of children and adolescents with CP; conducted in LMICs as per the World Bank classifications of low-income, lower-middle income and upper-middle income; and encompassing quantitative research designs/data.

To consolidate heterogeneous literature on health literacy, the Ophelia domains were adapted into a health literacy framework using the standardised HLQ to guide assessment.^16^ This review included any study covering at least one of the nine health literacy domains from the Ophelia framework, using relevant indicators based on the available literature, but did not use the original HLQ scoring system (see table 1 for proxy mapping).

**Table 1:** Proxy mapping

Ophelia domain		Original construct (HLQ)	Relevant indicators	# studies reported	Analysed
1	Feel understood and supported by healthcare provider	Has an established relationship with at least one healthcare provider who knows them well and who they trust to provide useful advice and information to assist them to understand information and make decisions about their health.	Having concerns understood	2	
			Having information relayed appropriately		
			Health professionals as sources of support		
2	Have sufficient information to manage my health	Feels confident that they have all the information that they need to live with and manage their condition and to make decisions.	Being up to date on best practice for care	3	
			Having a breadth of health information relating to malnutrition		
			Child development.		
3	Actively managing my health	Recognise the importance of health and are able to take responsibility for their own health. They proactively engage in their own care and make their own decision about their health. They make health a priority.	Aware of child’s need for service	6	ü
			Child health service utilisation		
4	Have social support for health	A person’s social system provides them with all the support they want or need for health.	Support for caregiving	7	ü
			Social isolation		
5	Appraise health information	Able to identify good information and reliable sources of information. They can resolve conflicting information by themselves or with help from others.	Understanding causes of condition	1	
6	Ability to actively engage with healthcare providers	Is proactive about their health and feels in control in relationships with healthcare providers. Is able to seek advice from additional healthcare providers when necessary. They keep going until they get what they want. Empowered.	Multiple health professionals as support	1	
7	Ability to navigate the healthcare system	Able to find out about services and supports so they get all their needs met. Able to advocate on their own behalf at the system and service level.	Health service access	5	ü
			Able to coordinate care		
			Scheduling and appointment compliance		
			Find information on available services		
8	Ability to find good health information	Is an information explorer. Actively uses a diverse range of sources to find information and is up to date.	Reported causes of illness	1	
9	Ability to understand information well enough to know what to do	Is able to understand all written information (including numerical information) in relation to their health and able to write appropriately on forms where required.	Education levels	14	ü
			Literacy/illiteracy		

HLQHealth Literacy QuestionnaireOpheliaOptimising Health Literacy and Access

Health literacy proxies for review included studies examining proxies for health literacy such as educational attainment, social capital, social support, self-efficacy, attitudes towards health, literacy skills, health information evaluation, communication skills, health information access, medication use, nutrition and physical activity, knowledge for health promotion, healthcare accessibility, awareness of rights and disease-specific knowledge.

Studies were excluded from this review if they were animal studies, systematic or scoping reviews, editorials, studies conducted in high-income countries, primary caregivers of children with intellectual or cognitive impairment, and qualitative studies.

The review was managed using Rayyan and EndNote V.21.2 software. After duplicates were removed, articles were screened based on title and abstract by three independent researchers (GLP, KA, RH) following the specified inclusion and exclusion criteria. Thereafter, researchers reviewed the full text of the articles to determine eligibility. In cases where there was disagreement regarding the inclusion of a study, a discussion was held, and a fourth reviewer (GK) was consulted to assess against the inclusion criteria until consensus was reached.

#### Data extraction

We developed and pilot-tested a data extraction template in Excel to systematically record information from the included studies. Data were extracted for each study independently by two reviewers (GLP and IJ). Extraction fields included the following: first author, publication date, study design, sample size (child and primary caregiver), country, primary caregiver sample composition, primary caregiver, mother, father and child educational attainment, household income, Gross Motor Function Classification System, institution or community-based recruitment, recruitment methods (convenience or key informant method), health literacy domains, determinants.

#### Data or statistical analysis

We conducted a thematic analysis for this review, systematically identifying, analysing and synthesising themes across a body of quantitative research studies. We have presented these data in tabular format. Due to the heterogeneity of the presented data, we were unable to do a pooled analysis. Table 1 outlines the proxy indicators for the Ophelia domains which emerged as part of our review. To ensure that the findings of this review were reflective of global LMICs, the authors opted for analysis and reporting to be conducted where a domain had reached a minimum of five publications.

#### Quality assessment of the literature

The included articles risk of bias was assessed using Study Quality Assessment Tool developed by the National Health, Lung, and Blood Institute. The overall findings from the risk of bias assessments are available in online supplemental table 2. Two researchers reviewed studies against the quality assessment framework (GLP, KA) and discrepancies were resolved via discussion until a conclusion was reached.

## Results

The systematic review yielded 2734 articles. Following title screening and abstract review, 40 articles were selected for full-text review, and of those, 15 articles were identified as eligible for inclusion (see figure 1). None of our included studies used a validated health literacy measurement tool, nor did they specifically measure health literacy. Studies only reported on individual components of the Ophelia domains (ie, Domain 3—managing my health, Domain 4—have social support for health, Domain 7—ability to navigate the healthcare system, Domain 9—ability to understand information well enough to know what to do). See table 1.

Researchers conducted a quality assessment for returned results—the range was between 1 and 14, and the average across included studies was 9.5/14. According to this quality assessment, most studies were of reasonable quality, and none were flagged for exclusion based on the assessment.

### Study characteristics

Included studies were from 11 LMICs, across 5 WHO defined regions, including Africa (Zimbabwe, Ghana, Benin, Nigeria), European region (Turkey), Southeast Asian (Bangladesh, Nepal), Western Pacific region (Cambodia, Indonesia) and the Eastern Mediterranean region (Jordan, Iran). There were no results returned for the Americas. The sample size of the included studies ranged between 40 and 2845 primary caregivers of children with CP. PCGs were predominately mothers. See online supplemental table 3 for a summary of demographic characteristics of participants of the included studies.

### Domain specific health literacy proxies

#### Domain 3—actively managing health

Awareness of health needs requires PCGs to assess the health and well-being of their child, feel motivated to address health concerns, and understand the potential of health services to promote quality of life.^18^ Therefore, in this review, we interpreted accessing health services as functional outcome for PCG health literacy. PCGs actively managing their child’s health would mean a child’s need would be the predominant determinant and they would have high levels of service utilisation and have consistent engagement with a breadth of services at regular intervals. Six studies (from six countries) returned results across two subdomains for actively managing health (table 2).

**Table 2:** Domain 3, actively managing health

Study	Country, number of participants (n)	Aware of child’s need for service	Child health service utilisation
Dambi and Jelsma^26^ 2014	Zimbabwe, n=46	30.0% PCG aware (ie, 23/75 PCGs completed the intervention)	X
Al Imam *et al*^10^ 2021	Bangladesh, n=2845	Commenced rehabilitation <5 years age: 69.6%	Ever received rehabilitation: 50.2%;AD: 4.7% of GMFCS III–V;PT: 44.6%*
Al Imam *et al*^19^ 2021	Bangladesh, n=2811	57.8% received/aware of service needs	Ever received rehabilitation: 50.2%;AD: 4.4%;PT: 44.9%;surgery: 0%*
	Ghana, n=277	81.8% received/aware about service needs	Ever received rehabilitation: 73.3%;AD: 36.8%;PT: 72.2%; surgery: 0%*
	Nepal, n=165	63.2% received/aware about service needs	Ever received rehabilitation: 54.2%;AD: 3.4%;PT: 37.6%; surgery: 5.6%*
	Indonesia, n=130	53.1% received/aware about service needs	Ever received rehabilitation: 33.8%;AD: 5.4%;PT: 28.7%; surgery: 0.8%*
Narayan *et al*^38^	Bangladesh, n=3890	x	Rehabilitation according to impairment: hearing: 39.8%, Speech: 53.9%, visual: 47.1%, intellectual: 51.4%,epilepsy: 56.3%
Almasari *et al*^20^ 2018	Jordan, n=114	Need for service as predictor of service utilisation p=0.003.	Children with higher service needs had lesser service receipt (Coeff: −0.28)
		PCG satisfaction with service as predictor of service utilisation p=<0.001.	
Saleh and Almasri^21^ 2016	Jordan, n=116	# of service receipt 0–3 years versus 13–18 years: only 1 type (1.7% vs 21.4%), 4–5 types (52.5% vs 28.6%)	Service receipt type:Medical: 65.1–73.7%, psychiatrist: 0%, PT: 90.4%,OT: 32.2%, ST: 7.8%,Orthotics: 25.2%, surgery consult: 22.9%

* Recalculated based on response on variables – —ever received rehabilitation and if yes then type of rehabilitation received.

AD, assistive device; OT, occupational therapyPT, physiotherapy

As reported in table 2, the average access rate of rehabilitation services for children with CP is 56.0%. Al Imam *et al*^19^ found a significant association between impairment severity and access: children with higher GMFCS scales (III–V) had a greater proportion of therapy receipt than GMFCS I–II across Bangladesh (52.0% vs 43%, p=0.001). Similar findings were reported in Ghana (79.8% vs 20.2%, p=0.225), Indonesia (9.1% vs 90.9%, p=0.118) and Nepal (79.8% vs 20.2%, p=0.106), although these were not significant.

However, in Jordan, children with greater need had lower service receipt. In fact, need for services was not the greatest predictor of access (p=0.03), rather parental satisfaction with services correlates with service access (p=<≤0.001).^20^ Similarly, the cohort in Saleh and Almasri from Jordan identified greater medical and rehabilitation needs in older children; however, this was not reflected in access rates. Saleh and Almasri^21^ found that children aged 13–18 years were less likely to receive rehabilitation services (85.7%, p value>0.05) compared with ages 7–12 years (100%, p value = >0.05), 4–6 years (100%, p value>0.05) and 0–3 years (93.2%, p value>0.05).^21^ In medical service access, 64.3% (p value = <0.05) of children aged between 13 and 18 years accessed medical services compared with 54.5% for 7–12-year-olds (p value = >0.05), 93.5% of 4–6-year-olds (p value = > 0.05) or 93.2% of 0–3-year-olds (p value = <0.05).

#### Domain 4—have social support for health

Domain 4 refers to the social support a PCG receives which promotes the health of their child with CP. Given the complexity and burden associated with caring for a child with disability, we assessed PCG’s structural and relational support. The former in this instance refers to the social resources available to parents, including the ability to access child care, assistance with appointments and caring duties, while the latter refers to value, breadth and depth of relationships in supporting PCGs.

Seven studies (six countries) reported components of domain 4 (see table 3).

**Table 3:** Domain 4—have social support for health

Study	Country, number of participants	Support for caregiving	Social isolation
Onwuakagba *et al*^22^	Nigeria, n=90	Really needs/would like help with issues with spouse (23.3%/27.8%)	Really need/would like friends who have a child like mine (41.1%/22.2%)
			Really need/would like someone to talk about my problems with (51.1%/25.6%)
		Really need/would like help with a babysitter so I can get away (43.3%/35.6%)	Caregiving is regularly/sometimes confining (40%/33.3%)
		Really need/would like help with childcare (72.2%/17.8%) Sufficient support from family (28.9%)	Would like help with how to deal with family (56.7%), friends or neighbours (62.3%)
		Sufficient support from friends (35.6%)	X
Mobarak *et al*^23^	Bangladesh, n=91	Helpful support received M=15.65/72 (SD 7.69)	X
Kenis-Coskun *et al*^25^	Turkey, n=107	96.3% married, 3.7% divorced	Social isolation/socialisation* (0–100) = mean 17.3 (SD 30.0)
		Burden of caregiving mean unmarried 54.10/100, married 46.50/100†	
Farajzadeh *et al*^24^	Iran, n=203	80.3% married, 19.7% single	Social functioning mean=52.8/100 (SD 22.4) (n=135)
Zuurmond *et al*^39^	Bangladesh, n=135	Family relationships quality mean=65.2/100 (SD 20)	64% satisfied with community
Strom et al^40^	Cambodia, n=40	x	Reports of stigma from neighbours (38.2%)
Sogbossi *et al*^11^	Benin, n=88	94.3% married, 5.7% widowed	X
		Spouse as sometimes helpful (10.2%) generally helpful (12.5%) very helpful (62.5%)	
		Friends as sometimes helpful (14.7%), generally helpful (0%), very helpful (0%)	
		Other children as sometimes helpful (9.0%), generally helpful (22.7), very helpful (14.7%)	
		Parents as sometimes helpful (29.5%), generally helpful (10.2%), very helpful (6.8%)	

* Range 0–100 higher score indicates better social functioning.

† Highest score 100, higher the score, the higher burden.

We identified marriage as a reservoir of support for primary caregivers in caring for a child with CP. This review suggests high rates of marriage reported by PCGs of children with CP in LMICs (Benin 94.3%, Iran 80.3%, Turkey 96.3%). PCGs in Benin identified marriage as the most profound form of social support for caring for children with CP.^11^ In Benin, 62.5% of respondents reported their spouse as very helpful, and 12.5% said their spouse was generally helpful in caring for a child with CP. This position is supported by findings from Turkey, in which the burden of caregiving was minimised in married women (burden mean for unmarried women—54.1/100 compared with 46.5/100 in married women, where the higher the score, the greater the burden).

Assessments of the breadth of the social network in LMICs suggest that support is deficient, as well as very radial. In Nigeria, 90% of PCGs ‘really need’ or ‘would like’ assistance with childcare, while 43.3% ‘really need’ a babysitter so that they can spend some time away.^22^ Parents of caregivers in Benin were only a moderate source of support at 6.8% very helpful, 10.2% generally helpful and 29.5% sometimes helpful. Comparatively, 14.7% of respondents claimed their friends were sometimes helpful, with 0% reporting friends as generally helpful or very helpful. In Bangladesh,^23^ the mean helpful support received was as low as 15.65/72 (SD 7.69). This suggests that even within friendship or familial circles, support for caring for children is not available. These findings suggest that there are insufficient numbers of people to assist with childcare.

PCGs of children with CP in LMICs are likely to experience social isolation associated with having a child with disability. Caregiving was reported as regularly or sometimes confining by 73.3% of PCGs in Nigeria.^22^ This follows from needing/liking friends with children like theirs (41.1%/22.2%) and help with dealing with family (56.7%) and friends/neighbours (62.3%). In measures of social functioning captured by Farajzadeh *et al* in Iran, PCGs of children with CP reported a mean of 52.8/100 (SD 22.4) (higher score indicates higher functioning) compared with a functioning score of 86.8/100 for PCGs of children without CP.^24^ Similarly, in Turkey, respondents reported their social health at 17.33/100 (SD 30.02—higher score indicating higher quality).^25^

#### Domain 7—ability to navigate the Healthcare system

Under Domain 7, we explored access rates, ability to coordinate care, appointment compliance and scheduling as well as PCG’s ability to find information to navigate the health system. Five studies (seven countries) reported components of Domain 7 (see table 4).

**Table 4:** Domain 7, Ability to navigate the healthcare system

Study	Country, number of participants (n)	Health service access (%)	Able to coordinate care	Scheduling and appointment compliance	Find information on available services
Al Imam *et al*^10^ 2021	Bangladesh n=2845		IB*: 44.1%		
Saleh and Almasri^21^	Jordan, n=116	Service use over previous 12 months†PT 90.4%Physician 73.7%Paediatrician 69.6%Neurologist 65.1%OT 32.2%Speech therapy <25%	Coordination between medical team and family: mean (SD) 3.0 (1.2) out of 4; 78.3% parents coordinate services by themselves; 47.4% rated the effort as ‘too much’	Regular visits for-Medical services: 40.4–49.5%, PT: 87.8%, OT: 32.2%, ST: 7%, orthotics: 17.4%, surgery consult: 15.6%	x
		Receipt of three to five services over previous 12 months†69.6%			
Onwuakagba *et al*^22^	Nigeria, n=90	X			Really need more help (74.4%)/would like some help (23.3%) finding programmes that can help my child
Sogbossi *et al*^11^	Benin, n=88	Support received from physician not available (60.2%), available (39.7%)			
		Support from gynaecologist or midwife following delivery not available (67%), available (32.9%)			
		Support received from social workers, physiotherapists, nurses not available (0%), available (99.9%)			
		Support received from NGOs for health—not available (4.5%), available (95.2%).			
		Support received from community-based rehab/health ministry—not available (0%), available (99.8%).			
Al Imam *et al*^19^	Bangladesh, n=2845	Not using PT services 47.5%	IB: 44.1%		Lack of awareness of service=84.2%
	Ghana, n=277	Not using PT services 26.7%	IB: 72.4%		Lack of awareness of service=68.9%
	Indonesia, n=130	Not using PT services 66.1%	IB: 49.4%		Lack of awareness=70.9%
	Nepal, n=165	Not using PT service 31.6%	IB: 16.5%		Lack of awareness=71.4%

* Institution-based recruitment.

† Children with cerebral palsy.

The **ability to navigate the healthcare system** (**Domain 7**) reflects on access and utilisation of available health services. Assessment of health service access across LMICs reveals a major deficit in overall use of services. In Bangladesh, 47.5% of respondents were not using available physiotherapy services, as well as 26.7% in Ghana, 66.1% in Indonesia and 31.6% in Nepal (Al Imam *et al*^19^). In Benin, 60.2% of respondents reported physicians and 67.0% of gynaecologists/midwives being unavailable.^11^ In Jordan, these figures were more positive—over 90% of respondents had accessed physiotherapy in the previous 12 months, with 69.6% having received three to five services. These high levels of uptake in Jordan may be representative of a highly educated cohort.^21^

A major barrier to navigating the health system was awareness of available services—84.2% of respondents in Bangladesh did not know where to access services. Awareness of available services was also low across Nepal (63.2% aware of services) and Indonesia (53.1% aware of services).^19^ Of the sites captured in Al Imam *et al*,^19^ Ghana was an outlier, where 81.8% of respondents reported being aware of services available to them. This is likely because Ghana used institution-based recruitment methods. Compared with transport (1.5%) and financial constraints (13.7%), most respondents within these studies cited an unawareness of availability (84.2%) as the main reason for underutilisation of available services.^19^

##### Schedule appointments and coordinate care

Several studies were institution-based, meaning their cohort had to some extent coordinated the care of their child. Dambi and Jelsma^26^ compared institution-based therapy and outreach services, and corresponding compliance rates and found that mean compliance was higher for outreach services (93.3%), compared with institutional services (72.3%). Similarly, they found that caregivers engaged with outreach services were more likely to participate in preventative health services, such as health promotion talks (six health promotion talks for outreach vs three in institution-based services). This may be explained by a lesser logistical burden on caregivers, making them more equipped to invest time in information exploration that would ultimately promote health literacy.

### Domain 9—ability to understand information well enough to know what to do

The high-level construct of Domain 9 is to understand written information well enough to know what to do. Therefore, literacy levels and educational attainment are good indicators of PCG proficiency in this domain. 14 studies (11 countries) reported literacy levels of PCGs (see table 5).

**Table 5:** Domain 9, understanding information well enough to know what to do

Study	Country	Number of participants	Literacy	Education PCG/mother
Dambi and Jelsma^26^	Zimbabwe	46	Literate 93%, illiterate 7%	Primary 9%, secondary 65%, tertiary 19%, none/illiterate 7%
Al Imam *et al*^10^	Bangladesh	2845	Literate 70%, illiterate 30%	No education 30.0% primary 39.8% secondary and above 30.1%
Sogbossi *et al*^11^	Benin	88	Literate 71.5%, illiterate 28.4%	No education 28.4%, primary 35.2%, secondary 26.1%, tertiary 10.2%Impact of CP on family—PCG not educated/primary education OR 4.07 (p value=0.048)
Almasri *et al*^20^	Jordan	112	Literate 100%, illiterate 0%	Primary 25.7%, secondary 37.2%, tertiary 37.1%
Al Imam *et al*^19^	Bangladesh	2845	Literate 69.5%, illiterate 29.8%	No education 30.2%, primary 40.0%secondary and above 30.3%
	Ghana	277	Literate 59.2%, illiterate 40.7%	No education 40.7%, primary 33.2%,secondary and above 25.9%
	Indonesia	130	Literate 87.6%, illiterate 12.3%	No education 12%Primary 49.2%Secondary and above 38.4%
	Nepal	179	Literate 65.2%, illiterate 38.8%	No education 38.8%, primary 32.9%,secondary and above 27.0%
Farajzadeh *et al*^24^	Iran	203	Literate 100% illiterate 0%	Primary/secondary 66%, tertiary 30%
Kenis-Coskun *et al*^25^	Turkey	107	Literate 100%, illiterate 0%	<Primary 3.5%Primary 32.9%Secondary 43.5%Tertiary 20%
Khan *et al*^41^	Bangladesh	92	Literate 62%, illiterate 38%	No education 38%Primary 31%Secondary and above 31%
Mobarak *et al*^23^	Bangladesh	91	Literate 58.2%, illiterate 38.4%	No education (38%)
Narayan *et al*^38^	Bangladesh	3820	Literate 76%, illiterate 24%	No education 24%Literate 76%
Onwuakagba *et al*^22^	Nigeria	90	Literate 100%, illiterate 0%	Secondary 61.1%Tertiary 38.9%
Power *et al*^42^	Bangladesh	154	Literate 44.2%, illiterate 55.8%	No education 55.8%Primary, secondary and tertiary 44.2%
Power *et al*^43^	Bangladesh	154	Literate 44.2%, illiterate 55.8%	No education 55.8%Primary, secondary and tertiary 44.2%
Saleh and Almasri^21^	Jordan	113	Literate 100%, illiterate 0%	<Secondary 25.7%Secondary 37.2%Tertiary 37.1%

PCGprimary caregiver

A substantial number of PCGs of children with CP in LMICs are illiterate, including up to 55% in Bangladesh, 38.8% in Nepal, 40.7% in Ghana, 28.4% in Benin, 12.3% in Indonesia and 7% in Zimbabwe. Several studies did not report less than primary school educational attainment (Nigeria, Turkey, Iran), or set illiteracy as an exclusion criterion (Jordan). Further, the study in Benin established that PCG education, be it no education or primary level, increased the likelihood of reported familial burden (OR 4.07, p value=0.048).^11^

Levels of educational attainment were diverse—PCGs that had no education were high in Bangladesh (up to 55.8%), Nepal (38.8%), Ghana (40.7%), Benin (28.4%) and Indonesia (12%). However, there were also significant proportions with postsecondary/tertiary qualifications: up to 44.3% in Bangladesh, 37.1% in Jordan, 38.9% in Nigeria, 20% in Turkey, 30% in Iran, 27% in Nepal, 25.9% in Ghana and 10.2% in Benin.

## Discussions

To the best of our knowledge, this is the first comprehensive analysis of health literacy skills and attributes of primary caregivers of children with CP in LMICs. Our systematic review reveals a significant gap in health literacy literature specifically tailored to the contexts of PCGs of children with CP in LMICs, highlighting the need for focused research and understanding in this area.

Our results indicate overall low service receipt among children with CP in LMICs, even in studies where participants were recruited or underwent institution-based interventions. Health service access issues are multifaceted and complex, but there is arguably a health literacy component that spans these cohorts—self-efficacy.^18 27^

Self-efficacy refers to the self-belief of personal efficacy in managing whatever challenges they may face.^28^ It is an important component of health literacy, insofar that self-efficacious individuals are more likely to accept a condition for what it is and be willing to take responsibility for its management.^29^ The results from Jordan which explored determinants of access, particularly those that accounted for multifactorial barriers,^20^ and still determined that greater child need did not equate with higher service receipt indicates an issue with PCG self-efficacy. The low self-efficacy hypothesis is further supported by declining service receipt for older children with CP. A number of studies reported declining service receipt with increasing age. The GLM CPR study by Al Imam at al^19^ covering Bangladesh, Nepal and Indonesia confirmed lesser service receipt for older children, as did the study by Almasari *et al*^20^ in Jordan. There would certainly be greater logistical burden getting older children to appointments; however, we also hypothesise that this may relate to declining levels of perceived self-efficacy. PCGs may be more hopeful about the potential to ‘cure’ or limit CP when the child is younger, and as the condition does not progress as hoped, they lose confidence in their management of it.^29 30^

The findings of low social support among PCGs have concerning implications for health and well-being because social support is a vital health resource, providing instrumental support, health information and encouragement of a healthier lifestyle. A robust social network helps with the practical aspects of raising a child with a disability, including physical, financial and emotional support. Marriage was identified as a major form of social support and had a positive relationship with minimising carer burden and contributing to overall health outcomes of children with CP. However, social support beyond spousal support was limited, with friends and even parents of PCGs providing minor aid. Across seven countries, every cohort experienced insufficient social support, leading to greater hardships in social standing and resources. Strong interpersonal relationships are essential for health as they act as conduits for health information, especially in settings with limited internet or media access.^31^ For PCGs of children with CP in LMICs, with limited social support, it is likely they are not receiving useful health information which would assist them managing the care of their child. An additional reason for why social networks are important is that they have been found to increase health seeking and health promoting behaviours for self and those under care.^32^ This is an important consideration, as addressing shortcomings in social support may promote greater health knowledge and behaviour among PCGs which would ultimately benefit the child’s health and well-being.

Our review indicates that many children with CP in LMICs have limited engagement with health services, affecting the timeliness of diagnosis, management and identification of associated impairments and complications.^1^ PCGs face a myriad of barriers to healthcare access in LMICs. However, beyond financial and resource constraints, a lack of awareness was cited as the number one factor impeding access to healthcare across multiple countries (Bangladesh, Ghana, Indonesia, Nepal,^19^ Nigeria^22^). Early identification of CP and intervention are paramount to promoting greater health outcomes, particularly in children below the age of five.^1^

The health system context significantly influences service navigability. Understanding health literacy includes the responsibilities of health professionals and policymakers.^33^ A comprehensive assessment of Domain 7 would need to account for the health literacy environment, which falls outside the scope of this review. However, our findings indicate the current norms in healthcare access, with further investigation needed to understand the low service engagement in LMICs.

Education and literacy are critical components of health literacy, with ‘significant independent association between education and health literacy’.^34^,^36^ Low educational attainment is a risk factor for low health literacy and poor health outcomes.^34^ Health literacy can mediate the health risk associated with low educational attainment. In Bangladesh, education was significantly associated with low rehabilitation service uptake (p≤0.001), though more research is needed to understand why.^10^ Our findings show a varied picture for educational attainment across LMICs, with a major portions of PCGs with little to no education, while others are highly educated. Some studies used institution-based recruitment, potentially introducing selection bias, and some excluded illiterate parents. Despite these limitations, we found education as a significant determinant of health literacy and a modifiable risk for poor health outcomes, presenting an opportunity for further exploration.

## Limitations

One limitation of our study is the potential introduction of inaccuracies when using proxies in place of direct health literacy measures. This can lead to discrepancies between the intended outcome and the actual data obtained, thereby reducing the reliability of the review’s findings. Our finding confirms and extends previous work of Zaidman *et al*,^15^ whose LMIC findings were based on interviews and national surveys capturing educational attainment and literacy rates. Although a limitation, our review is novel and the first of its kind, offering valuable insights into a under-researched topic.

Also, considering the dearth of studies directly measuring health literacy in this cohort, we recognise the limitation that arises from the heterogeneity of data collected across studies, which precluded the ability to conduct a meta-analysis. Variations in study aims and designs, participant demographics, interventions and outcome measures made it challenging to statistically combine results in this review.

## Conclusion

Our review revealed a critical gap; no studies actively measured health literacy or used appropriate tools to assess it among PCGs of children with CP in LMICs. This highlights a significant knowledge gap and a missed opportunity to address the unique needs of this vulnerable population group. This study fills a crucial void in the literature by providing a comprehensive review of the current state of knowledge, identifying key challenges such as social support, service navigation, self-efficacy and educational attainment. These gaps warrant further investigation. Future research should focus on developing and validating fit-for-purpose health literacy tools for PCGs of children with CP in LMICs. Moreover, interventions aimed at enhancing health literacy should be tailored to the specific cultural and local healthcare contexts in LMICs.

## supplementary material

10.1136/bmjopen-2024-091679 online supplemental file 1

## Data Availability

Data sharing not applicable as no datasets generated and/or analysed for this study.
