# Supplementary material for: Health literacy of primary caregivers of children with cerebral palsy in low- and middle-income countries: a systematic review
Source: BMJ Open. 2025 Mar 3;15(3):e091679. doi: 10.1136/bmjopen-2024-091679 (PMC11877220; doi:10.1136/bmjopen-2024-091679)
Supplement: online supplemental file 1 [file bmjopen-15-3-s001.docx]

*Supplementary table 1 – Ovid Medline search strategy*

**MEDLINE: Heath literacy – Caregivers of children with a disability – FINAL (as at 19.01.24)**

**Notes: FINAL – additional disability terms removed.**

Database: Ovid MEDLINE(R) ALL <1946 to January 18, 2024>

Search Strategy:

--------------------------------------------------------------------------------

1 exp Parents/ (145931)

2 exp Grandparents/ (765)

3 exp Caregivers/ (51753)

4 exp Legal Guardians/ (3908)

5 (parent$ or mother$ or father$ or grandparent$ or grandmother$ or grandfather$ or caregiver$ or guardian$).tw. (822488)

6 1 or 2 or 3 or 4 or 5 (863420)

7 exp Disabled Persons/ (74367)

8 (disabilit$ or disable$).tw. (267691)

9 exp Developmental Disabilities/ (22502)

10 (development$ adj1 (disabilit$ or delay$ or disorder$ or disease$ or syndrom$ or condition$ or deficit$)).tw. (58224)

11 exp Brain Injuries/ (84058)

12 (brain$ adj3 injur$).tw. (91825)

13 exp Motor Disorders/ (1097)

14 (motor adj2 (disorder$ or deficit$)).tw. (18330)

15 exp Neurodevelopmental Disorders/ (212845)

16 exp "Congenital, Hereditary, and Neonatal Diseases and Abnormalities"/ (1383548)

17 ((neurodevelopment$ or genetic$) adj2 (disabilit$ or delay$ or disorder$ or disease$ or syndrom$ or condition$ or deficit$)).tw. (90587)

18 exp Hypoxia, Brain/ (14487)

19 ((hypoxi$ or ischaemi$ or ischemi$) adj1 encephal$).tw. (5521)

20 exp Cerebral Palsy/ (24055)

21 (cerebral adj1 pals$).tw. (27300)

22 CP.tw. (70466)

23 exp Muscular Dystrophies/ (30269)

24 (muscular adj1 dystroph$).tw. (27424)

25 (physical$ adj1 impair$).tw. (4951)

26 7 or 8 or 9 or 10 or 11 or 12 or 13 or 14 or 15 or 16 or 17 or 18 or 19 or 20 or 21 or 22 or 23 or 24 or 25 (2119621)

27 exp Infant/ (1265190)

28 exp Child/ (2183116)

29 exp Adolescent/ (2232487)

30 (baby or babies or infant$ or toddler$ or child$ or pediatric$ or paediatric$ or adolescen$ or teenager$ or youth$).tw. (2454840)

31 27 or 28 or 29 or 30 (4654401)

32 6 and 26 and 31 (112728)

33 exp Developing Countries/ (81683)

34 (developing adj1 (countr$ or nation or nations)).tw. (76904)

35 (low adj2 income adj2 (countr$ or nation or nations)).tw. (12140)

36 (middle adj2 income adj2 (countr$ or nation or nations)).tw. (36209)

37 LMIC.tw. (3764)

38 (third adj1 world).tw. (3197)

39 (less adj1 developed adj1 (country or countries or nation or nations or setting$)).tw. (1633)

40 ((country or countries or nation or nations or setting$) and (resource$ adj3 (limit$ or constrain$))).tw. (24069)

41 33 or 34 or 35 or 36 or 37 or 38 or 39 or 40 (192021)

42 32 and 41 (1435)

43 exp Health Literacy/ (9819)

44 ((health$ or ehealth$) adj5 (literac$ or literate)).tw. (16160)

45 exp Perception/ (484648)

46 (health adj3 (percept$ or perceiv$ or insight$ or observation or observations or interpret$)).tw. (34172)

47 exp Educational Status/ (62936)

48 (education$ adj3 (status or attain$ or achievement$ or accomplish$ or complet$)).tw. (43490)

49 exp social capital/ (1705)

50 (social$ adj3 (capital$ or invest$ or resource$ or cognition)).tw. (25915)

51 exp Self Efficacy/ (24684)

52 (self adj3 (efficacy or effective$ or useful$ or abilit$ or able or complacen$ or affirm$)).tw. (63073)

53 ((oral$ or litera$) adj3 (skill$ or proficienc$ or abilit$)).tw. (6327)

54 exp Reading/ (25994)

55 (read or reads or reading).tw. (206272)

56 exp Writing/ (42055)

57 (write or writing).tw. (44931)

58 exp Literacy/ (1762)

59 (literate or literac$).tw. (32803)

60 exp Health Knowledge, Attitudes, Practice/ (127699)

61 (health adj3 (knowledge or aware$ or understand$)).tw. (41526)

62 (health adj3 (attitude$ or belief$)).tw. (18133)

63 exp Health Behavior/ (364536)

64 (health adj3 (behaviour$ or behavior$ or experience$ or practice$)).tw. (120842)

65 exp Patient Rights/ (83817)

66 (right$ adj3 (patient$ or health$ or aware$ or conscious$)).tw. (42215)

67 exp Medication Adherence/ (26124)

68 exp Patient Compliance/ (86232)

69 (adhere$ or comply or compliance).tw. (382706)

70 exp Consumer Health Information/ (13817)

71 ((health or information) adj3 (material$ or brochure$ or handout$ or leaflet$ or poster$ or sign$)).tw. (73054)

72 exp Information Seeking Behavior/ (3260)

73 (information adj3 (seek$ or need$)).tw. (33562)

74 exp Patient Education as Topic/ (88742)

75 (patient adj3 educat$).tw. (31676)

76 exp Access to Information/ (8224)

77 (information$ adj3 (availab$ or utili$ or suppl$ or barrier$)).tw. (105546)

78 (health adj3 (skill$ or abilit$ or proficien$ or competen$)).tw. (14598)

79 exp Communication/ (371334)

80 (health adj3 communicat$).tw. (12924)

81 exp Adaptation, Psychological/ (140840)

82 (psycholog$ adj3 (adapt$ or adjust$)).tw. (6031)

83 ((coping or cope or handl$) adj3 (strateg$ or abilit$)).tw. (29997)

84 ((perception$ or perceive$) adj3 burden$).tw. (2865)

85 (health adj3 challenge$).tw. (28798)

86 (health adj3 adapt$).tw. (4126)

87 empower$.tw. (40269)

88 exp Problem Solving/ (30765)

89 (problem$ adj3 solv$).tw. (72859)

90 exp Resilience, Psychological/ (8755)

91 resilien$.tw. (62648)

92 (facilitator$ or barrier$ or determinant$ or prequisite$ or requisite$ or precondition$ or obstruct$).tw. (1080198)

93 exp Stakeholder Participation/ (4188)

94 (health adj3 (partnership$ or involve$ or contribut$)).tw. (30881)

95 exp Patient Advocacy/ (24243)

96 (advoca$ or activism$ or activist$).tw. (98029)

97 or/43-96 (3686556)

98 42 and 97 (493)

99 exp animals/ not humans.sh. (5188347)

100 98 not 99 (493)

*CINAHL*

**CINAHL via EBSCO: Health literacy – Caregivers of children with a disability – FINAL (as at 20.01.24)**

**Notes: FINAL – additional disability terms removed.**

**Search History**

| **Search ID#** | **Search Terms** | **Results** |
| --- | --- | --- |
| S98 | S41 AND S97  **Limiters** - Exclude MEDLINE records; Human | 859 |
| S97 | S41 AND S97 | 1,740 |
| S96 | S42 OR S43 OR S44 OR S45 OR S46 OR S47 OR S48 OR S49 OR S50 OR S51 OR S52 OR S53 OR S54 OR S55 OR S56 OR S57 OR S58 OR S59 OR S60 OR S61 OR S62 OR S63 OR S64 OR S65 OR S66 OR S67 OR S68 OR S69 OR S70 OR S71 OR S72 OR S73 OR S74 OR S75 OR S76 OR S77 OR S78 OR S79 OR S80 OR S81 OR S82 OR S83 OR S84 OR S85 OR S86 OR S87 OR S88 OR S89 OR S90 OR S91 OR S92 OR S93 OR S94 OR S95 OR S96 | 1,455,914 |
| S95 | advoca* OR activism* OR activist* | 68,761 |
| S94 | (MH "Patient Advocacy") | 14,880 |
| S93 | (health W3 (partnership* OR involve* OR contribut*)) | 10,606 |
| S92 | (MH "Stakeholder Participation") | 3,282 |
| S91 | facilitator* OR barrier* OR determinant* OR prequisite* OR requisite* OR precondition* OR obstruct* | 297,830 |
| S90 | resilien* | 24,494 |
| S89 | problem* W3 solv* | 21,109 |
| S88 | (MH "Problem Solving+") | 13,909 |
| S87 | empower* | 37,241 |
| S86 | health W3 adapt* | 1,386 |
| S85 | health W3 challenge* | 10,205 |
| S84 | ((perception* OR perceive*) W3 burden*) | 1,643 |
| S83 | ((coping OR cope OR handl*) W3 (strateg* OR abilit*)) | 13,908 |
| S82 | (psycholog* W3 (adapt* OR adjust*)) | 2,924 |
| S81 | (MH "Adaptation, Psychological+") | 42,115 |
| S80 | health W3 communicat* | 5,513 |
| S79 | (MH "Communication+") | 335,279 |
| S78 | (health W3 (skill* OR abilit* OR proficien* OR competen*)) | 6,342 |
| S77 | (information* W3 (availab* OR utili* OR suppl* OR barrier*)) | 20,039 |
| S76 | (MH "Access to Information+") | 27,380 |
| S75 | patient W3 educat* | 91,091 |
| S74 | (MH "Patient Education+") | 84,640 |
| S73 | (information W3 (seek* OR need*)) | 25,649 |
| S72 | (MH "Information Seeking Behavior") | 5,146 |
| S71 | ((health OR information) W3 (material* OR brochure* OR handout* OR leaflet* OR poster* OR sign*)) | 15,273 |
| S70 | (MH "Consumer Health Information+") | 20,841 |
| S69 | adhere* OR comply OR compliance | 165,945 |
| S68 | (MH "Patient Compliance+") | 58,253 |
| S67 | (MH "Medication Compliance") | 23,962 |
| S66 | (right* W3 (patient* OR health* OR aware* OR conscious*)) | 8,284 |
| S65 | (MH "Patient Rights+") | 23,442 |
| S64 | (health W3 (behaviour* OR behavior* OR experience* OR practice*)) | 104,464 |
| S63 | (MH "Health Behavior+") | 122,176 |
| S62 | (health W3 (attitude* OR belief*)) | 23,869 |
| S61 | (health W3 (knowledge OR aware* OR understand*)) | 51,618 |
| S60 | (MH "Attitude to Health+") | 182,485 |
| S59 | (MH "Health Knowledge") | 38,618 |
| S58 | literate OR literac* | 29,197 |
| S57 | (MH "Literacy") | 6,087 |
| S56 | write OR writing | 39,951 |
| S55 | (MH "Writing+") | 25,757 |
| S54 | read OR reads OR reading | 59,335 |
| S53 | (MH "Reading+") | 16,748 |
| S52 | ((oral* OR litera*) W3 (skill* OR proficienc* OR abilit*)) | 3,620 |
| S51 | (self W3 (efficacy OR effective* OR useful* OR abilit* OR able OR complacen* OR affirm*)) | 41,920 |
| S50 | (MH "Self-Efficacy") | 27,429 |
| S49 | (social* W3 (capital* OR invest* OR resource* OR cognition)) | 11,175 |
| S48 | (MH "Social Capital") | 3,099 |
| S47 | (education* W3 (status OR attain* OR achievement* OR accomplish* OR complet*)) | 59,970 |
| S46 | (MH "Educational Status") | 48,156 |
| S45 | (health W3 (percept* OR perceiv* OR insight* OR observation OR observations OR interpret*)) | 10,717 |
| S44 | (MH "Perception+") | 94,776 |
| S43 | ((health* OR ehealth*) W5 (literac* OR literate)) | 12,715 |
| S42 | (MH "Health Literacy") | 7,193 |
| S41 | S31 AND S40 | 4,389 |
| S40 | S32 OR S33 OR S34 OR S35 OR S36 OR S37 OR S38 OR S39 | 547,822 |
| S39 | ((country OR countries OR nation OR nations OR setting*) AND (resource* W3 (limit* OR constrain*)) | 5,315 |
| S38 | less W1 developed W1 (country OR countries OR nation OR nations OR setting* | 547,245 |
| S37 | third W1 world | 615 |
| S36 | LMIC | 3,774 |
| S35 | (middle W2 income W2 (countr* OR nation OR nations)) | 16,214 |
| S34 | (low W2 income W2 (countr* OR nation OR nations)) | 16,878 |
| S33 | developing W1 (countr* OR nation OR nations) | 33,077 |
| S32 | (MH "Developing Countries") | 19,975 |
| S31 | S6 AND S25 AND S30 | 41,480 |
| S30 | S26 OR S27 OR S28 OR S29 | 1,490,032 |
| S29 | baby OR babies OR infant* OR toddler* OR child* OR pediatric* OR paediatric* OR adolescen* OR teenager* OR youth* | 1,490,032 |
| S28 | (MH "Adolescence+") | 613,130 |
| S27 | (MH "Child+") | 767,644 |
| S26 | (MH "Infant+") | 292,491 |
| S25 | S7 OR S8 OR S9 OR S10 OR S11 OR S12 OR S13 OR S14 OR S15 OR S16 OR S17 OR S18 OR S19 OR S20 OR S21 OR S22 OR S23 OR S24 | 512,483 |
| S24 | physical* W1 impair* | 1,994 |
| S23 | muscular W1 dystroph* | 5,071 |
| S22 | (MH "Muscular Dystrophy+") | 4,582 |
| S21 | CP | 17,673 |
| S20 | cerebral W1 pals* | 18,063 |
| S19 | (MH "Cerebral Palsy") | 14,044 |
| S18 | ((hypoxi* OR ischaemi* OR ischemi*) W1 encephal*) | 1,835 |
| S17 | (MH "Hypoxia, Brain+") | 2,590 |
| S16 | ((neurodevelopment* OR genetic*) W2 (disabilit* OR delay* OR disorder* OR disease* OR syndrom* OR condition* OR deficit*)) | 15,272 |
| S15 | (MH "Congenital, Hereditary, and Neonatal Diseases and Abnormalities+") | 225,247 |
| S14 | (motor W2 (disorder* OR deficit*)) | 6,250 |
| S13 | (MH "Motor Skills Disorders") | 2,764 |
| S12 | brain* W3 injur* | 39,734 |
| S11 | (MH "Brain Injuries+") | 33,889 |
| S10 | development* W1 (disabilit* OR delay* OR disorder* OR disease* OR syndrom* OR condition* OR deficit*) | 27,141 |
| S9 | (MH "Developmental Disabilities") | 11,631 |
| S8 | disabilit* OR disable* | 207,517 |
| S7 | (MH "Persons with Disabilities+") | 67,923 |
| S6 | S1 OR S2 OR S3 OR S4 OR S5 | 402,415 |
| S5 | parent* OR mother* OR father* OR grandparent* OR grandmother* OR grandfather* OR caregiver* OR guardian* | 401,236 |
| S4 | (MH "Guardianship, Legal+") | 2,076 |
| S3 | (MH "Caregivers") | 44,208 |
| S2 | (MH "Grandparents") | 2,540 |
| S1 | (MH "Parents+") | 120,579 |

*Scopus*

**SCOPUS: Health literacy – Caregivers of children with a disability – FINAL (as at 20.01.24)**

**Notes: FINAL – additional disability terms removed.**

( ( TITLE-ABS-KEY ( ( ( health* OR ehealth* ) W/5 ( literac* OR literate ) ) ) ) OR ( TITLE-ABS-KEY ( ( health W/3 ( percept* OR perceiv* OR insight* OR observation OR observations OR interpret* ) ) ) ) OR ( TITLE-ABS-KEY ( ( education* W/3 ( status OR attain* OR achievement* OR accomplish* OR complet* ) ) ) ) OR ( TITLE-ABS-KEY ( ( social* W/3 ( capital* OR invest* OR resource* OR cognition ) ) ) ) OR ( TITLE-ABS-KEY ( ( self W/3 ( efficacy OR effective* OR useful* OR abilit* OR able OR complacen* OR affirm* ) ) ) ) OR ( TITLE-ABS-KEY ( ( ( oral* OR litera* ) W/3 ( skill* OR proficienc* OR abilit* ) ) ) ) OR ( TITLE-ABS-KEY ( read OR reads OR reading OR write OR writing OR literate OR literac* ) ) OR ( TITLE-ABS-KEY ( ( health W/3 ( knowledge OR aware* OR understand* ) ) ) ) OR ( TITLE-ABS-KEY ( ( health W/3 ( attitude* OR belief* ) ) ) ) OR ( TITLE-ABS-KEY ( ( health W/3 ( behaviour* OR behavior* OR experience* OR practice* ) ) ) ) OR ( TITLE-ABS-KEY ( ( right* W/3 ( patient* OR health* OR aware* OR conscious* ) ) ) ) OR ( TITLE-ABS-KEY ( ( ( health OR information ) W/3 ( material* OR brochure* OR handout* OR leaflet* OR poster* OR sign* ) ) ) ) OR ( TITLE-ABS-KEY ( ( information W/3 ( seek* OR need* ) ) ) ) OR ( TITLE-ABS-KEY ( ( information* W/3 ( availab* OR utili* OR suppl* OR barrier* ) ) ) ) OR ( TITLE-ABS-KEY ( ( health W/3 ( skill* OR abilit* OR proficien* OR competen* ) ) ) ) OR ( TITLE-ABS-KEY ( ( health W/3 communicat* ) OR ( patient W/3 educat* ) ) ) OR ( TITLE-ABS-KEY ( ( psycholog* W/3 ( adapt* OR adjust* ) ) ) ) OR ( TITLE-ABS-KEY ( ( ( coping OR cope OR handl* ) W/3 ( strateg* OR abilit* ) ) ) ) OR ( TITLE-ABS-KEY ( ( ( perception* OR perceive* ) W/3 burden* ) ) ) OR ( TITLE-ABS-KEY ( ( health W/3 challenge* ) OR ( health W/3 adapt* ) ) ) OR ( TITLE-ABS-KEY ( facilitator* OR barrier* OR determinant* OR prequisite* OR requisite* OR precondition* OR problem* W/3 solv* ) ) OR ( TITLE-ABS-KEY ( ( health W/3 ( partnership* OR involve* OR contribut* ) ) ) ) OR ( TITLE-ABS-KEY ( adhere* OR comply OR compliance OR empower* OR resilien* OR obstruct* OR advoca* OR activism* OR activist* ) ) ) AND ( ( TITLE-ABS-KEY ( developing W/1 ( countr* OR nation OR nations ) ) ) OR ( TITLE-ABS-KEY ( ( low W/2 income W/2 ( countr* OR nation OR nations ) ) ) ) OR ( TITLE-ABS-KEY ( ( middle W/2 income W/2 ( countr* OR nation OR nations ) ) ) ) OR ( TITLE-ABS-KEY ( lmic OR ( third W/1 world ) ) ) OR ( TITLE-ABS-KEY ( less W/1 developed W/1 ( country OR countries OR nation OR nations OR setting* ) ) ) OR ( TITLE-ABS-KEY ( ( ( country OR countries OR nation OR nations OR setting* ) AND ( resource* W/3 ( limit* OR constrain* ) ) ) ) ) ) AND ( ( TITLE-ABS-KEY ( parent* OR mother* OR father* OR grandparent* OR grandmother* OR grandfather* OR caregiver* OR guardian* ) ) AND ( ( TITLE-ABS-KEY ( disabilit* OR disable* ) ) OR ( TITLE-ABS-KEY ( development* W/1 ( disabilit* OR delay* OR disorder* OR disease* OR syndrom* OR condition* OR deficit* ) ) ) OR ( TITLE-ABS-KEY ( brain* W/3 injur* ) ) OR ( TITLE-ABS-KEY ( ( motor W/2 ( disorder* OR deficit* ) ) ) ) OR ( TITLE-ABS-KEY ( ( ( neurodevelopment* OR genetic* ) W/2 ( disabilit* OR delay* OR disorder* OR disease* OR syndrom* OR condition* OR deficit* ) ) ) ) OR ( TITLE-ABS-KEY ( ( ( hypoxi* OR ischaemi* OR ischemi* ) W/1 encephal* ) ) ) OR ( TITLE-ABS-KEY ( ( cerebral W/1 pals* ) OR cp ) ) OR ( TITLE-ABS-KEY ( ( muscular W/1 dystroph* ) OR ( physical* W/1 impair* ) ) ) ) AND ( TITLE-ABS-KEY ( baby OR babies OR infant* OR toddler* OR child* OR pediatric* OR paediatric* OR adolescen* OR teenager* OR youth* ) ) )

44 ( ( TITLE-ABS-KEY ( ( ( health* OR ehealth* ) W/5 ( literac* OR literate ) ) ) ) OR ( TITLE-ABS-KEY ( ( health W/3 ( percept* OR perceiv* OR insight* OR observation OR observations OR interpret* ) ) ) ) OR ( TITLE-ABS-KEY ( ( education* W/3 ( status OR attain* OR achievement* OR accomplish* OR complet* ) ) ) ) OR ( TITLE-ABS-KEY ( ( social* W/3 ( capital* OR invest* OR resource* OR cognition ) ) ) ) OR ( TITLE-ABS-KEY ( ( self W/3 ( efficacy OR effective* OR useful* OR abilit* OR able OR complacen* OR affirm* ) ) ) ) OR ( TITLE-ABS-KEY ( ( ( oral* OR litera* ) W/3 ( skill* OR proficienc* OR abilit* ) ) ) ) OR ( TITLE-ABS-KEY ( read OR reads OR reading OR write OR writing OR literate OR literac* ) ) OR ( TITLE-ABS-KEY ( ( health W/3 ( knowledge OR aware* OR understand* ) ) ) ) OR ( TITLE-ABS-KEY ( ( health W/3 ( attitude* OR belief* ) ) ) ) OR ( TITLE-ABS-KEY ( ( health W/3 ( behaviour* OR behavior* OR experience* OR practice* ) ) ) ) OR ( TITLE-ABS-KEY ( ( right* W/3 ( patient* OR health* OR aware* OR conscious* ) ) ) ) OR ( TITLE-ABS-KEY ( ( ( health OR information ) W/3 ( material* OR brochure* OR handout* OR leaflet* OR poster* OR sign* ) ) ) ) OR ( TITLE-ABS-KEY ( ( information W/3 ( seek* OR need* ) ) ) ) OR ( TITLE-ABS-KEY ( ( information* W/3 ( availab* OR utili* OR suppl* OR barrier* ) ) ) ) OR ( TITLE-ABS-KEY ( ( health W/3 ( skill* OR abilit* OR proficien* OR competen* ) ) ) ) OR ( TITLE-ABS-KEY ( ( health W/3 communicat* ) OR ( patient W/3 educat* ) ) ) OR ( TITLE-ABS-KEY ( ( psycholog* W/3 ( adapt* OR adjust* ) ) ) ) OR ( TITLE-ABS-KEY ( ( ( coping OR cope OR handl* ) W/3 ( strateg* OR abilit* ) ) ) ) OR ( TITLE-ABS-KEY ( ( ( perception* OR perceive* ) W/3 burden* ) ) ) OR ( TITLE-ABS-KEY ( ( health W/3 challenge* ) OR ( health W/3 adapt* ) ) ) OR ( TITLE-ABS-KEY ( facilitator* OR barrier* OR determinant* OR prequisite* OR requisite* OR precondition* OR problem* W/3 solv* ) ) OR ( TITLE-ABS-KEY ( ( health W/3 ( partnership* OR involve* OR contribut* ) ) ) ) OR ( TITLE-ABS-KEY ( adhere* OR comply OR compliance OR empower* OR resilien* OR obstruct* OR advoca* OR activism* OR activist* ) ) ) AND ( ( TITLE-ABS-KEY ( developing W/1 ( countr* OR nation OR nations ) ) ) OR ( TITLE-ABS-KEY ( ( low W/2 income W/2 ( countr* OR nation OR nations ) ) ) ) OR ( TITLE-ABS-KEY ( ( middle W/2 income W/2 ( countr* OR nation OR nations ) ) ) ) OR ( TITLE-ABS-KEY ( lmic OR ( third W/1 world ) ) ) OR ( TITLE-ABS-KEY ( less W/1 developed W/1 ( country OR countries OR nation OR nations OR setting* ) ) ) OR ( TITLE-ABS-KEY ( ( ( country OR countries OR nation OR nations OR setting* ) AND ( resource* W/3 ( limit* OR constrain* ) ) ) ) ) ) AND ( ( TITLE-ABS-KEY ( parent* OR mother* OR father* OR grandparent* OR grandmother* OR grandfather* OR caregiver* OR guardian* ) ) AND ( ( TITLE-ABS-KEY ( disabilit* OR disable* ) ) OR ( TITLE-ABS-KEY ( development* W/1 ( disabilit* OR delay* OR disorder* OR disease* OR syndrom* OR condition* OR deficit* ) ) ) OR ( TITLE-ABS-KEY ( brain* W/3 injur* ) ) OR ( TITLE-ABS-KEY ( ( motor W/2 ( disorder* OR deficit* ) ) ) ) OR ( TITLE-ABS-KEY ( ( ( neurodevelopment* OR genetic* ) W/2 ( disabilit* OR delay* OR disorder* OR disease* OR syndrom* OR condition* OR deficit* ) ) ) ) OR ( TITLE-ABS-KEY ( ( ( hypoxi* OR ischaemi* OR ischemi* ) W/1 encephal* ) ) ) OR ( TITLE-ABS-KEY ( ( cerebral W/1 pals* ) OR cp ) ) OR ( TITLE-ABS-KEY ( ( muscular W/1 dystroph* ) OR ( physical* W/1 impair* ) ) ) ) AND ( TITLE-ABS-KEY ( baby OR babies OR infant* OR toddler* OR child* OR pediatric* OR paediatric* OR adolescen* OR teenager* OR youth* ) ) )

408 results

43 ( TITLE-ABS-KEY ( ( ( health* OR ehealth* ) W/5 ( literac* OR literate ) ) ) ) OR ( TITLE-ABS-KEY ( ( health W/3 ( percept* OR perceiv* OR insight* OR observation OR observations OR interpret* ) ) ) ) OR ( TITLE-ABS-KEY ( ( education* W/3 ( status OR attain* OR achievement* OR accomplish* OR complet* ) ) ) ) OR ( TITLE-ABS-KEY ( ( social* W/3 ( capital* OR invest* OR resource* OR cognition ) ) ) ) OR ( TITLE-ABS-KEY ( ( self W/3 ( efficacy OR effective* OR useful* OR abilit* OR able OR complacen* OR affirm* ) ) ) ) OR ( TITLE-ABS-KEY ( ( ( oral* OR litera* ) W/3 ( skill* OR proficienc* OR abilit* ) ) ) ) OR ( TITLE-ABS-KEY ( read OR reads OR reading OR write OR writing OR literate OR literac* ) ) OR ( TITLE-ABS-KEY ( ( health W/3 ( knowledge OR aware* OR understand* ) ) ) ) OR ( TITLE-ABS-KEY ( ( health W/3 ( attitude* OR belief* ) ) ) ) OR ( TITLE-ABS-KEY ( ( health W/3 ( behaviour* OR behavior* OR experience* OR practice* ) ) ) ) OR ( TITLE-ABS-KEY ( ( right* W/3 ( patient* OR health* OR aware* OR conscious* ) ) ) ) OR ( TITLE-ABS-KEY ( ( ( health OR information ) W/3 ( material* OR brochure* OR handout* OR leaflet* OR poster* OR sign* ) ) ) ) OR ( TITLE-ABS-KEY ( ( information W/3 ( seek* OR need* ) ) ) ) OR ( TITLE-ABS-KEY ( ( information* W/3 ( availab* OR utili* OR suppl* OR barrier* ) ) ) ) OR ( TITLE-ABS-KEY ( ( health W/3 ( skill* OR abilit* OR proficien* OR competen* ) ) ) ) OR ( TITLE-ABS-KEY ( ( health W/3 communicat* ) OR ( patient W/3 educat* ) ) ) OR ( TITLE-ABS-KEY ( ( psycholog* W/3 ( adapt* OR adjust* ) ) ) ) OR ( TITLE-ABS-KEY ( ( ( coping OR cope OR handl* ) W/3 ( strateg* OR abilit* ) ) ) ) OR ( TITLE-ABS-KEY ( ( ( perception* OR perceive* ) W/3 burden* ) ) ) OR ( TITLE-ABS-KEY ( ( health W/3 challenge* ) OR ( health W/3 adapt* ) ) ) OR ( TITLE-ABS-KEY ( facilitator* OR barrier* OR determinant* OR prequisite* OR requisite* OR precondition* OR problem* W/3 solv* ) ) OR ( TITLE-ABS-KEY ( ( health W/3 ( partnership* OR involve* OR contribut* ) ) ) ) OR ( TITLE-ABS-KEY ( adhere* OR comply OR compliance OR empower* OR resilien* OR obstruct* OR advoca* OR activism* OR activist* ) ) 6,549,280 results

42 ( TITLE-ABS-KEY ( developing W/1 ( countr* OR nation OR nations ) ) ) OR ( TITLE-ABS-KEY ( ( low W/2 income W/2 ( countr* OR nation OR nations ) ) ) ) OR ( TITLE-ABS-KEY ( ( middle W/2 income W/2 ( countr* OR nation OR nations ) ) ) ) OR ( TITLE-ABS-KEY ( lmic OR ( third W/1 world ) ) ) OR ( TITLE-ABS-KEY ( less W/1 developed W/1 ( country OR countries OR nation OR nations OR setting* ) ) ) OR ( TITLE-ABS-KEY ( ( ( country OR countries OR nation OR nations OR setting* ) AND ( resource* W/3 ( limit* OR constrain* ) ) ) ) ) 443,699 results

41 TITLE-ABS-KEY ( adhere* OR comply OR compliance OR empower* OR resilien* OR obstruct* OR advoca* OR activism* OR activist* ) 2,294,174 results

40 TITLE-ABS-KEY ( ( health W/3 ( partnership* OR involve* OR contribut* ) ) ) 61,663 results

39 TITLE-ABS-KEY ( facilitator* OR barrier* OR determinant* OR prequisite* OR requisite* OR precondition* OR problem* W/3 solv* ) 1,191,287 results

38 TITLE-ABS-KEY ( ( health W/3 challenge* ) OR ( health W/3 adapt* ) ) 58,371 results

37 TITLE-ABS-KEY ( ( ( perception* OR perceive* ) W/3 burden* ) ) 4,106 results

36 TITLE-ABS-KEY ( ( ( coping OR cope OR handl* ) W/3 ( strateg* OR abilit* ) ) ) 73,434 results

35 TITLE-ABS-KEY ( ( psycholog* W/3 ( adapt* OR adjust* ) ) ) 106,616 results

34 TITLE-ABS-KEY ( ( health W/3 communicat* ) OR ( patient W/3 educat* ) ) 213,097 results

33 TITLE-ABS-KEY ( ( health W/3 ( skill* OR abilit* OR proficien* OR competen* ) ) ) 28,917 results

32 TITLE-ABS-KEY ( ( information* W/3 ( availab* OR utili* OR suppl* OR barrier* ) ) ) 290,134 results

31 TITLE-ABS-KEY ( ( information W/3 ( seek* OR need* ) ) ) 134,167 results

30 TITLE-ABS-KEY ( ( ( health OR information ) W/3 ( material* OR brochure* OR handout* OR leaflet* OR poster* OR sign* ) ) ) 226,348 results

29 TITLE-ABS-KEY ( ( right* W/3 ( patient* OR health* OR aware* OR conscious* ) ) ) 99,327 results

28 TITLE-ABS-KEY ( ( health W/3 ( behaviour* OR behavior* OR experience* OR practice* ) ) )

367,382 results

27 TITLE-ABS-KEY ( ( health W/3 ( attitude* OR belief* ) ) ) 342,034 results

26 TITLE-ABS-KEY ( ( health W/3 ( knowledge OR aware* OR understand* ) ) ) 188,440 results

25 TITLE-ABS-KEY ( read OR reads OR reading OR write OR writing OR literate OR literac* )

1,261,944 results

24 TITLE-ABS-KEY ( ( ( oral* OR litera* ) W/3 ( skill* OR proficienc* OR abilit* ) ) ) 25,965 results

23 TITLE-ABS-KEY ( ( self W/3 ( efficacy OR effective* OR useful* OR abilit* OR able OR complacen* OR affirm* ) ) ) 155,191 results

22 TITLE-ABS-KEY ( ( social* W/3 ( capital* OR invest* OR resource* OR cognition ) ) ) 130,920 results

21 TITLE-ABS-KEY ( ( education* W/3 ( status OR attain* OR achievement* OR accomplish* OR complet* ) ) ) 201,198 results

20 TITLE-ABS-KEY ( ( health W/3 ( percept* OR perceiv* OR insight* OR observation OR observations OR interpret* ) ) ) 59,164 results

19 TITLE-ABS-KEY ( ( ( health* OR ehealth* ) W/5 ( literac* OR literate ) ) ) 29,704 results

18 TITLE-ABS-KEY ( ( ( country OR countries OR nation OR nations OR setting* ) AND ( resource* W/3 ( limit* OR constrain* ) ) ) ) 39,116 results

17 TITLE-ABS-KEY ( less W/1 developed W/1 ( country OR countries OR nation OR nations OR setting* ) )7,778 results

16 TITLE-ABS-KEY ( lmic OR ( third W/1 world ) ) 25,045 results

15 TITLE-ABS-KEY ( ( middle W/2 income W/2 ( countr* OR nation OR nations ) ) ) 53,693 results

14 TITLE-ABS-KEY ( ( low W/2 income W/2 ( countr* OR nation OR nations ) ) ) 61,446 results

13 TITLE-ABS-KEY ( developing W/1 ( countr* OR nation OR nations ) ) 339,612 results

12 ( TITLE-ABS-KEY ( parent* OR mother* OR father* OR grandparent* OR grandmother* OR grandfather* OR caregiver* OR guardian* ) ) AND ( ( TITLE-ABS-KEY ( disabilit* OR disable* ) ) OR ( TITLE-ABS-KEY ( development* W/1 ( disabilit* OR delay* OR disorder* OR disease* OR syndrom* OR condition* OR deficit* ) ) ) OR ( TITLE-ABS-KEY ( brain* W/3 injur* ) ) OR ( TITLE-ABS-KEY ( ( motor W/2 ( disorder* OR deficit* ) ) ) ) OR ( TITLE-ABS-KEY ( ( ( neurodevelopment* OR genetic* ) W/2 ( disabilit* OR delay* OR disorder* OR disease* OR syndrom* OR condition* OR deficit* ) ) ) ) OR ( TITLE-ABS-KEY ( ( ( hypoxi* OR ischaemi* OR ischemi* ) W/1 encephal* ) ) ) OR ( TITLE-ABS-KEY ( ( cerebral W/1 pals* ) OR cp ) ) OR ( TITLE-ABS-KEY ( ( muscular W/1 dystroph* ) OR ( physical* W/1 impair* ) ) ) ) AND ( TITLE-ABS-KEY ( baby OR babies OR infant* OR toddler* OR child* OR pediatric* OR paediatric* OR adolescen* OR teenager* OR youth* ) 69,070 results

11 TITLE-ABS-KEY ( baby OR babies OR infant* OR toddler* OR child* OR pediatric* OR paediatric* OR adolescen* OR teenager* OR youth* ) 6,033,538 results

10 ( TITLE-ABS-KEY ( disabilit* OR disable* ) ) OR ( TITLE-ABS-KEY ( development* W/1 ( disabilit* OR delay* OR disorder* OR disease* OR syndrom* OR condition* OR deficit* ) ) ) OR ( TITLE-ABS-KEY ( brain* W/3 injur* ) ) OR ( TITLE-ABS-KEY ( ( motor W/2 ( disorder* OR deficit* ) ) ) ) OR ( TITLE-ABS-KEY ( ( ( neurodevelopment* OR genetic* ) W/2 ( disabilit* OR delay* OR disorder* OR disease* OR syndrom* OR condition* OR deficit* ) ) ) ) OR ( TITLE-ABS-KEY ( ( ( hypoxi* OR ischaemi* OR ischemi* ) W/1 encephal* ) ) ) OR ( TITLE-ABS-KEY ( ( cerebral W/1 pals* ) OR cp ) ) OR ( TITLE-ABS-KEY ( ( muscular W/1 dystroph* ) OR ( physical* W/1 impair* ) ) )

1,480,472 results

9 TITLE-ABS-KEY ( ( muscular W/1 dystroph* ) OR ( physical* W/1 impair* ) ) 56,197 results

8 TITLE-ABS-KEY ( ( cerebral W/1 pals* ) OR cp ) 244,333 results

7 TITLE-ABS-KEY ( ( ( hypoxi* OR ischaemi* OR ischemi* ) W/1 encephal* ) ) 12,910 results

6 TITLE-ABS-KEY ( ( ( neurodevelopment* OR genetic* ) W/2 ( disabilit* OR delay* OR disorder* OR disease* OR syndrom* OR condition* OR deficit* ) ) ) 322,823 results

5 TITLE-ABS-KEY ( ( motor W/2 ( disorder* OR deficit* ) ) ) 30,635 results

4 TITLE-ABS-KEY ( brain* W/3 injur* ) 196,243 results

3 TITLE-ABS-KEY ( development* W/1 ( disabilit* OR delay* OR disorder* OR disease* OR syndrom* OR condition* OR deficit* ) )180,588 results

2 TITLE-ABS-KEY ( disabilit* OR disable* ) 568,566 results

1 TITLE-ABS-KEY ( parent* OR mother* OR father* OR grandparent* OR grandmother* OR grandfather* OR caregiver* OR guardian* ) 1,579,174 results

**Web of Science Core Collection: Health literacy – Caregivers of children with a disability – FINAL (as at 20.01.24)**

**Notes: FINAL – additional disability terms removed.**

Science Citation Index Expanded (SCI-EXPANDED)-1900-present

Social Sciences Citation Index (SSCI)-1956-present

Arts & Humanities Citation Index (AHCI)-1975-present

Conference Proceedings Citation Index – Science (CPCI-S)-1990-present

Conference Proceedings Citation Index – Social Science & Humanities (CPCI-SSH)-1990-present

Emerging Sources Citation Index (ESCI)-2005-present

Current Chemical Reactions (CCR-EXPANDED)-1985-present

Index Chemicus (IC)-1993-present

Date Run: Sat Jan 20 2024 19:17:42 GMT+1100 (Australian Eastern Daylight Time)

# Searches:

1: parent* OR mother* OR father* OR grandparent* OR grandmother* OR grandfather* OR caregiver* OR guardian* (Topic) Results: 1180441

2: disabilit* OR disable* (Topic) Results: 408961

3: development* NEAR/1 (disabilit* OR delay* OR disorder* OR disease* OR syndrom* OR condition* OR deficit*) (Topic) Results: 115947

4: brain* NEAR/3 injur* (Topic) Results: 140010

5: TS=((motor NEAR/2 (disorder* OR deficit*))) Results: 22794

6: TS=(((neurodevelopment* OR genetic*) NEAR/2 (disabilit* OR delay* OR disorder* OR disease* OR syndrom* OR condition* OR deficit*))) Results: 116847

7: TS=(((hypoxi* OR ischaemi* OR ischemi*) NEAR/1 encephal*)) Results: 7569

8: TS=((cerebral NEAR/1 pals*) OR CP) Results: 196767

9: TS=((muscular NEAR/1 dystroph*) OR (physical* NEAR/1 impair*) ) Results: 47662

10: #2 OR #3 OR #4 OR #5 OR #6 OR #7 OR #8 OR #9 Results: 980446

11: TS=(baby OR babies OR infant* OR toddler* OR child* OR pediatric* OR paediatric* OR adolescen* OR teenager* OR youth*) Results: 3410538

12: #11 AND #10 AND #1 Results: 43038

13: TS=(developing NEAR/1 (countr* OR nation OR nations)) Results: 237497

14: TS=((low W/2 income NEAR/2 (countr* OR nation OR nations))) Results: 0

15: TS=((low NEAR/2 income NEAR/2 (countr* OR nation OR nations))) Results: 49753

16: TS=((middle NEAR/2 income NEAR/2 (countr* OR nation OR nations))) Results: 46408

17: TS=(LMIC OR (third NEAR/1 world) ) Results: 18019

18: TS=(less NEAR/1 developed NEAR/1 (country OR countries OR nation OR nations OR setting*)) Results: 7266

19: TS=(((country OR countries OR nation OR nations OR setting*) AND (resource* NEAR/3 (limit* OR constrain*)))) Results: 32900

20: TS=(((health* OR ehealth*) NEAR/5 (literac* OR literate))) Results: 23694

21: TS=((health NEAR/3 (percept* OR perceiv* OR insight* OR observation OR observations OR interpret*))) Results: 50256

22: TS=((education* NEAR/3 (status OR attain* OR achievement* OR accomplish* OR complet*))) Results: 73836

23: TS=((social* NEAR/3 (capital* OR invest* OR resource* OR cognition))) Results: 107393

24: TS=((self NEAR/3 (efficacy OR effective* OR useful* OR abilit* OR able OR complacen* OR affirm*))) Results: 146747

25: TS=(((oral* OR litera*) NEAR/3 (skill* OR proficienc* OR abilit*))) Results: 20209

26: TS=(read OR reads OR reading OR write OR writing OR literate OR literac*)Results: 1127422

27: TS=((health NEAR/3 (knowledge OR aware* OR understand*))) Results: 65985

28: TS=((health NEAR/3 (attitude* OR belief*))) Results: 27183

29: TS=((health NEAR/3 (behaviour* OR behavior* OR experience* OR practice*)))Results: 169202

30: TS=((right* NEAR/3 (patient* OR health* OR aware* OR conscious*))) Results: 65492

31: TS=(((health OR information) NEAR/3 (material* OR brochure* OR handout* OR leaflet* OR poster* OR sign*))) Results: 164267

32: TS=((information NEAR/3 (seek* OR need*))) Results: 91133

33: TS=((information* NEAR/3 (availab* OR utili* OR suppl* OR barrier*))) Results: 202254

34: TS=((health NEAR/3 (skill* OR abilit* OR proficien* OR competen*))) Results: 22014

35: TS=((health NEAR/3 communicat*) OR (patient W/3 educat*)) Results: 22387

36: TS=((psycholog* NEAR/3 (adapt* OR adjust*))) Results: 14321

37: TS=(((coping OR cope OR handl*) NEAR/3 (strateg* OR abilit*))) Results: 60249

38: TS=(((perception* OR perceive*) NEAR/3 burden*)) Results: 4015

39: TS=((health NEAR/3 challenge*) OR (health NEAR/3 adapt*)) Results: 45372

40: TS=((facilitator* OR barrier* OR determinant* OR prequisite* OR requisite* OR precondition* OR problem*) NEAR/3 solv*) Results: 660101

41: TS=((health NEAR/3 (partnership* OR involve* OR contribut*))) Results: 46078

42: TS=(adhere* OR comply OR compliance OR empower* OR resilien* OR obstruct* OR advoca* OR activism* OR activist*) Results: 1511568

43: #42 OR #41 OR #40 OR #39 OR #38 OR #37 OR #36 OR #35 OR #34 OR #33 OR #32 OR #31 OR #30 OR #29 OR #28 OR #27 OR #26 OR #25 OR #24 OR #23 OR #22 OR #21 OR #20 Results: 4304601

44: #13 OR #14 OR #15 OR #16 OR #17 OR #18 OR #19 Results: 328418

45: #43 AND #44 AND #12 Results: 281

**EMBASE: Heath literacy – Caregivers of children with a disability – FINAL (as at 19.01.24)**

**Notes: FINAL – additional disability terms removed.**

Database: Embase Classic+Embase <1947 to 2024 January 18>

Search Strategy:

--------------------------------------------------------------------------------

1 exp parent/ (331402)

2 exp grandparent/ (5383)

3 exp caregiver/ (118298)

4 exp legal guardian/ (1289)

5 (parent$ or mother$ or father$ or grandparent$ or grandmother$ or grandfather$ or caregiver$ or guardian$).tw. (1118661)

6 1 or 2 or 3 or 4 or 5 (1217658)

7 exp disabled person/ (60402)

8 (disabilit$ or disable$).tw. (387003)

9 exp developmental disorder/ (63083)

10 (development$ adj1 (disabilit$ or delay$ or disorder$ or disease$ or syndrom$ or condition$ or deficit$)).tw. (82517)

11 exp brain injury/ (231485)

12 (brain$ adj3 injur$).tw. (130784)

13 exp motor dysfunction/ (1039043)

14 (motor adj2 (disorder$ or deficit$)).tw. (27308)

15 exp congenital disorder/ (1788344)

16 ((neurodevelopment$ or genetic$) adj2 (disabilit$ or delay$ or disorder$ or disease$ or syndrom$ or condition$ or deficit$)).tw. (130646)

17 exp brain hypoxia/ (13023)

18 ((hypoxi$ or ischaemi$ or ischemi$) adj1 encephal$).tw. (8362)

19 exp cerebral palsy/ (49951)

20 (cerebral adj1 pals$).tw. (41943)

21 CP.tw. (101973)

22 exp muscular dystrophy/ (59245)

23 (muscular adj1 dystroph$).tw. (40349)

24 (physical$ adj1 impair$).tw. (7324)

25 7 or 8 or 9 or 10 or 11 or 12 or 13 or 14 or 15 or 16 or 17 or 18 or 19 or 20 or 21 or 22 or 23 or 24 (3527787)

26 exp infant/ (1336145)

27 exp child/ (3570253)

28 exp adolescent/ (1955537)

29 (baby or babies or infant$ or toddler$ or child$ or pediatric$ or paediatric$ or adolescen$ or teenager$ or youth$).tw. (3375148)

30 26 or 27 or 28 or 29 (5374999)

31 6 and 25 and 30 (140781)

32 exp developing country/ (103924)

33 (developing adj1 (countr$ or nation or nations)).tw. (99864)

34 (low adj2 income adj2 (countr$ or nation or nations)).tw. (15385)

35 (middle adj2 income adj2 (countr$ or nation or nations)).tw. (42474)

36 LMIC.tw. (5376)

37 (third adj1 world).tw. (3887)

38 (less adj1 developed adj1 (country or countries or nation or nations or setting$)).tw. (1840)

39 ((country or countries or nation or nations or setting$) and (resource$ adj3 (limit$ or constrain$))).tw. (32226)

40 32 or 33 or 34 or 35 or 36 or 37 or 38 or 39 (237028)

41 31 and 40 (1767)

42 exp health literacy/ (20910)

43 ((health$ or ehealth$) adj5 (literac$ or literate)).tw. (20749)

44 exp perception/ (544203)

45 (health adj3 (percept$ or perceiv$ or insight$ or observation or observations or interpret$)).tw. (41483)

46 exp educational status/ (144158)

47 (education$ adj3 (status or attain$ or achievement$ or accomplish$ or complet$)).tw. (59427)

48 exp social capital/ (4117)

49 (social$ adj3 (capital$ or invest$ or resource$ or cognition)).tw. (31087)

50 exp self concept/ (258746)

51 (self adj3 (efficacy or effective$ or useful$ or abilit$ or able or complacen$ or affirm$)).tw. (75389)

52 ((oral$ or litera$) adj3 (skill$ or proficienc$ or abilit$)).tw. (7462)

53 exp reading/ (64072)

54 (read or reads or reading).tw. (282866)

55 exp writing/ (49969)

56 (write or writing).tw. (60171)

57 exp literacy/ (6991)

58 (literate or literac$).tw. (39860)

59 exp attitude to health/ (135362)

60 (health adj3 (knowledge or aware$ or understand$)).tw. (51575)

61 (health adj3 (attitude$ or belief$)).tw. (21530)

62 exp health behavior/ (513412)

63 (health adj3 (behaviour$ or behavior$ or experience$ or practice$)).tw. (142573)

64 exp patient right/ (204407)

65 (right$ adj3 (patient$ or health$ or aware$ or conscious$)).tw. (67983)

66 exp medication compliance/ (48514)

67 exp patient compliance/ (195772)

68 (adhere$ or comply or compliance).tw. (582759)

69 exp consumer health information/ (4311)

70 ((health or information) adj3 (material$ or brochure$ or handout$ or leaflet$ or poster$ or sign$)).tw. (99022)

71 exp information seeking/ (6143)

72 (information adj3 (seek$ or need$)).tw. (43649)

73 exp patient education/ (127524)

74 (patient adj3 educat$).tw. (50606)

75 exp access to information/ (31348)

76 (information$ adj3 (availab$ or utili$ or suppl$ or barrier$)).tw. (123464)

77 (health adj3 (skill$ or abilit$ or proficien$ or competen$)).tw. (17834)

78 exp interpersonal communication/ (847673)

79 (health adj3 communicat$).tw. (15790)

80 exp psychological adjustment/ (10968)

81 (psycholog$ adj3 (adapt$ or adjust$)).tw. (7679)

82 ((coping or cope or handl$) adj3 (strateg$ or abilit$)).tw. (37763)

83 ((perception$ or perceive$) adj3 burden$).tw. (4044)

84 (health adj3 challenge$).tw. (33554)

85 (health adj3 adapt$).tw. (4929)

86 empower$.tw. (53478)

87 exp problem solving/ (44777)

88 (problem$ adj3 solv$).tw. (89270)

89 exp psychological resilience/ (11233)

90 resilien$.tw. (69117)

91 (facilitator$ or barrier$ or determinant$ or prequisite$ or requisite$ or precondition$ or obstruct$).tw. (1461442)

92 exp stakeholder engagement/ (8967)

93 (health adj3 (partnership$ or involve$ or contribut$)).tw. (37824)

94 exp patient advocacy/ (24979)

95 (advoca$ or activism$ or activist$).tw. (136119)

96 42 or 43 or 44 or 45 or 46 or 47 or 48 or 49 or 50 or 51 or 52 or 53 or 54 or 55 or 56 or 57 or 58 or 59 or 60 or 61 or 62 or 63 or 64 or 65 or 66 or 67 or 68 or 69 or 70 or 71 or 72 or 73 or 74 or 75 or 76 or 77 or 78 or 79 or 80 or 81 or 82 or 83 or 84 or 85 or 86 or 87 or 88 or 89 or 90 or 91 or 92 or 93 or 94 or 95 (5246139)

97 41 and 96 (703)

98 (animal$ not human$).sh. (5330486)

99 97 not 98 (700)

***************************

*Supplementary table 2 – Study Quality Assessment Tool by the National Health, Lung and Blood Institute*

|  | Q1 | Q2 | Q3 | Q4 | Q5 | Q6 | Q7 | Q8 | Q9 | Q10 | Q11 | Q12 | Q13 | Q14 |  |
| --- | --- | --- | --- | --- | --- | --- | --- | --- | --- | --- | --- | --- | --- | --- | --- |
| Al Imam et al 2021 | Y | Y | Y | Y | N | Y | Y | Y | Y | N | Y | N | N/A | Y | 10 |
| Sogbossi et al 2022 | Y | Y | Y | Y | N | Y | Y | N | Y | N | Y | N/A | N/A | N | 8 |
| Ström et al 2012 | Y | Y | N/A | Y | N | Y | Y | N | Y | N | Y | NA | NA | N | 7 |
| Zuurmond et al 2015 | Y | Y | Y | Y | N | Y | Y | Y | Y | N | Y | NA | NA | Y | 10 |
| Almasri et al 2018 | Y | Y | Y | Y | Y | Y | Y | Y | Y | N | Y | NA | NA | Y | 11 |
| Al Imam et al 2021 B | Y | Y | Y | Y | Y | Y | Y | Y | Y | N | Y | NA | NA | Y | 11 |
| Farajzadeh et al 2019 | Y | Y | NA | Y | Y | Y | Y | Y | Y | N | Y | NA | NA | Y | 10 |
| Kenis-Coskun et al 2020 | Y | N | Y | Y | Y | Y | Y | Y | Y | N | Y | NA | NA | Y | 10 |
| Khan et al 1998 | Y | Y | Y | Y | N | Y | Y | NA | Y | Y | Y | NA | Y | N | 10 |
| Mobarak et al 2000 | Y | Y | Y | Y | N | Y | Y | N | N | N | Y | NA | Y | N | 8 |
| Narayan et al 2023 | Y | Y | Y | Y | Y | Y | Y | Y | Y | N | Y | NA | Y | Y | 13 |
| Onwuakagba et al 2022 | Y | Y | Y | N | N | Y | Y | N | N | N | Y | NA | Y | Y | 8 |

**Scoring: Yes = 1 point, No or NA = 0**

1. Was the research question or objective in this paper clearly stated?

2. Was the study population clearly specified and defined?

3. Was the participation rate of eligible persons at least 50%?

4. Were all the subjects selected or recruited from the same or similar populations (including the same time period)? Were inclusion and exclusion criteria for being in the study prespecified and applied uniformly to all participants?

5. Was a sample size justification, power description, or variance and effect estimates provided?

6. For the analyses in this paper, were the exposure(s) of interest measured prior to the outcome(s) being measured?

7. Was the timeframe sufficient so that one could reasonably expect to see an association between exposure and outcome if it existed?

8. For exposures that can vary in amount or level, did the study examine different levels of the exposure as related to the outcome (e.g., categories of exposure, or exposure measured as continuous variable)?

9. Were the exposure measures (independent variables) clearly defined, valid, reliable, and implemented consistently across all study participants?

10. Was the exposure(s) assessed more than once over time?

11. Were the outcome measures (dependent variables) clearly defined, valid, reliable, and implemented consistently across all study participants?

12. Were the outcome assessors blinded to the exposure status of participants?

13. Was loss to follow-up after baseline 20% or less?

14. Were key potential confounding variables measured and adjusted statistically for their impact on the relationship between exposure(s) and outcome(s)?

Supplementary Table 3 - *Demographic characteristics of PCGs of children with CP of the included studies*

| Author | Country | Number of participants | PCG Composition | PCG Sex/age* | PCG Education | PCG Employment/ occupation | Monthly family income (USD) * |
| --- | --- | --- | --- | --- | --- | --- | --- |
| Dambi & Jelsma[18] | Zimbabwe | 46 | Mother 83.0% Grandmother 11.0%  Sibling 7.0% | F 93.5% | Illiterate 7.0% Primary 9.0%, Secondary 65.0%, Tertiary 19.0%, | Employed:  Informally 30.0% Formally 9.0%  Unemployed 61.0% | x |
| Al Imam et al [10] | Bangladesh | 2845 | Mother 100.0% | F 100.0% | Illiterate 30.0% Primary 39.9%  Secondary & above 30.1% | X | $6-118: 67.5%  >$119: 32.3% |
| Sogbossi et al [11] | Benin | 88 | Mother 100.0% | F 100%/ 36.42 (7.64) | Illiterate 28.4%, Primary 35.2%, Secondary and above 36.3% | Employed -Informally 70.4%  Formally 5.6%  Unemployed 22.7% /  Housewife 22.7% Retailer 36.3% Artisans 20.4% Trader 14.7% Formally employed 5.6% | x |
| Ström et al [19] | Cambodia | 40 | Mother 90.0%, Father 2.5%, Other 7.5% | F 90.0% M 2.5% | X | X | X |
| Zuurmond et al [20] | Bangladesh | 135 | Mother 88%, Other 10% | F 88% | X | X | X |
| Almasri et al [21] | Jordan | 116 | Mother 96.5% | F 96.5% / 31.07 (6.78) | Primary 25.7% Secondary 37.2% Tertiary 37.1% | Employed  Full time 6.4% Part time 0.9% Unemployed (92.7%). | ≤$423: 47.0% $424-$1411: 47.0%  >$1411: 6.0% |
| Al Imam et al [22] | Bangladesh | 2845 | Mother 99.3% | F 100% | Illiterate 30.0% Primary 39.7%, Secondary & above 30.2% | X | $0-50: 6.8%  $51-100: 54.5%  >$100: 37.4% |
|  | Ghana | 277 | Mother 100% | F 100% | Illiterate 40.7% Primary 33.2% Secondary & above 25.9% | X | $0-50: 47.2%  $51-100: 27.7%  >$100: 24.9% |
|  | Indonesia | 130 | Mother 100% | F 100% | Illiterate 12.3% Primary 49.2% Secondary & above 46.1%. | X | $0-50: 80.0%,  $51-100: 11.5%  $>100 8.4% |
|  | Nepal | 182 | Mother 93.4% | F 93.4% | Illiterate 36.2% Primary 30.7% Secondary & above 25.2% | X | $0-50: 17.3%  $51-100: 34.0%  $>100: 48.6% |
| Farajzadeh et al [23] | Iran | 203 | Mother 100% | F 100% /34.48 (6.74) | Primary/ secondary 70.0%, Tertiary 30% | Employed 34.0%  Unemployed 66.0% | x |
| Kenis-Coskun et al [24] | Turkey | 107 | Mother 75.7% Father 24.2% | F 81  M 26/  35.83  (7.07) | Literate 3.5% Primary 32.9% Secondary 43.5% Tertiary 20% | Employed 32.7%  Unemployed 67.2% | x |
| Khan et al [25] | Bangladesh | 92 | Mother 100% | F 100% | Illiterate 38.0% Primary 31.0% Secondary and above 31.0% | X | x |
| Mobarak et al [26] | Bangladesh | 91 | Mother 100% | F 100% / 25.8(5.4) | Illiterate 38%  Literate 62% | x | Mean  $51 (SD $68) |
| Narayan et al [27] | Bangladesh | 3820 | Mother 100% | x | Illiterate 21.8% Literate 78.2% | X | <$146: 90.9%  $146-243: 6.1%  >$243: 2.0% |
| Onwuakagba et al [28] | Nigeria | 90 | Mother 67.8%  Father 32.2% | F 67.8%  M 32.2% / Range  18-30 48.8%, 31-50 51.1% | Secondary and above 100% | x / Banker 16.7%  Nurse 20.0% Teacher 35.6%  Trader 27.8% | x |
| Power et al [29] | Bangladesh | 154 | Mother 76%, Father 13.6% Other 9.6% | F 81.8% M 18.1%  /39y 9m (9y 9m) | Illiterate 55.8% Literate 44.2% | x | Median  $54 (IQR $45) |
| Power, Galea et al [30] | Bangladesh | 154 | x | F 81.8% M 18.1%  39y 9m (9y, 9m) | Illiterate 55.8%  Literate 44.2% | X | Median  $54 (IQR $45) |

* all local currencies have been converted to USD [rate as of 8 May 2024]
