# Supplementary material for: Health literacy of primary caregivers of children with cerebral palsy in low- and middle-income countries: a systematic review
Source: BMJ Open. 2025 Mar 3;15(3):e091679. doi: 10.1136/bmjopen-2024-091679 (PMC11877220; doi:10.1136/bmjopen-2024-091679)
Supplement: Supplementary file 2 [file bmjopen-2024-091679.reviewer_comments.pdf]

PEER REVIEW HISTORY

BMJ Open publishes all reviews undertaken for accepted manuscripts. Reviewers are asked to complete a checklist review form and are provided with free text boxes to elaborate on their assessment. These free text comments are reproduced below.

ARTICLE DETAILS

Title (Provisional)

Health literacy of primary caregivers of children with cerebral palsy in low-and middle-income countries: a systematic review

Authors

Perrins, Genevieve Lilian; King, Catherine; Azhdari, Kousar; Jahan, Israt; Power, Rosalie; Hashan, Rashidul; Badawi, Nadia; Khandaker, Gulam

---

VERSION 1 - REVIEW

Reviewer 1

Name Osako, Miho

Affiliation Department of Neurology, Tokyo Metropolitan Kita Medical and Rehabilitation Center for the Disabled, 1-2-3 Jujodai, Kita-ku, Tokyo 114-0033, Japan, Neurology

Date 05-Sep-2024

COI I have no competing interests.

This paper provides valuable insight because it investigates an area that has not been well researched: the health literacy of caregivers of people with cerebral palsy in low-and middle-income countries. The results are described separately in the domain of health literacy, and this study will be a helpful reference for future research on what interventions should be used. However, several things need to be improved. I have noted the recommendation below.

Introduction, Methods, and Results section
-There needs to be an introduction of the "Ophelia" process, what it is, and what its aim is, in the Introduction section or the Methods section with references. This is because no information was provided about this framework. There are many different measures of health literacy, but how did authors come to use the HLQ of Ophelia framework, and what advantages does it have over other measures for examining caregivers' health literacy in middle- and low-income countries?
-The flowchart in Figure 1 should be moved to the first part of the Results section.
-Table 1 should be shown in the Results section after Figure 1.
-I could not find Supplementary Table 2 in the draft.

Discussion section

-Is there a difference between well-studied domains (domains 3, 4, 7, and 9) and less-studied domains (domains 1, 2, 5, 6, and 8), such as differences in the evaluation feasibility? If so, could you explain?

Others

-Please attach the PRISMA checklist.

-I did not understand the search formula in Supplementary Table 1. I think it is usually indicated by AND, NOT OR, etc., but is what you showed us a different way to indicate it?

**Reviewer**	**2**
**Name**	**Fluss, J.**
**Affiliation**	**Univ Hosp Geneva**
**Date**	**08-Sep-2024**
**COI**	**none**

I have accepted to review this manuscript having explored both the topic of literacy and SES, as well as the field of CP.

The manuscript is very well writen and brings very important message for any professionals involve in the care of CP children in the setting of family living in or coming from LMICs.

It would have been maybe meaningful for the authors (and for the readership) to have a better explanation of the Ophelia framework, and eventually to discuss it a bit more within the introduction.

As many potential readers are presumably coming from high income countries, and encounter in their practice more and more immigrant children with CP from LiMCs, it could be of interest to discuss briefly this issue (has it been studied?).

practical points:

the authors should clarify why they do not cover all domains in the result section (unclear).

VERSION 1 - AUTHOR RESPONSE

Reviewer 1:

This paper provides valuable insight because it investigates an area that has not been well researched: the health literacy of caregivers of people with cerebral palsy in low-and middle income countries. The results are described separately in the domain of health literacy, and this study will be a helpful reference for future research on what interventions should be used. However, several things need to be improved. I have noted the recommendation below.

Introduction, Methods, and Results section
-There needs to be an introduction of the "Ophelia" process, what it is, and what its aim is, in the Introduction section or the Methods section with references. This is because no information was provided about this framework. There are many different measures of health literacy, but how did authors come to use the HLQ of Ophelia framework, and what advantages does it have over other measures for examining caregivers' health literacy in middle- and low-income countries?

Our response: Thank you for bringing this to our attention. We have amended the methods section to include a background on the Ophelia process and the way in which the domains were used as part of this systematic review. Please see pages 3 and 4.
-The flowchart in Figure 1 should be moved to the first part of the Results section.
Our response: Amended and update (see revised Fig 1 in the manuscript).
-Table 1 should be shown in the Results section after Figure 1.
Our response: Now we have placed Table 1 in the results section after Figure 1.
-I could not find Supplementary Table 2 in the draft.
Our response: The supplementary tables have been amended and resubmitted, and their reference in the article revised. Thank you.
Discussion section
-Is there a difference between well-studied domains (domains 3, 4, 7, and 9) and less-studied domains (domains 1, 2, 5, 6, and 8), such as differences in the evaluation feasibility? If so, could you explain?

Our response: Thank you for this helpful comment. We have amended to include a statement relating to inclusion within analysis. See section 'Data or statistical analysis' in tracked changes.

Others
-Please attach the PRISMA checklist.
Our response: We have completed and attached the PRISMA checklist.

-I did not understand the search formula in Supplementary Table 1. I think it is usually indicated by AND, NOT OR, etc., but is what you showed us a different way to indicate it?
Our response: Thank you for flagging this. Our search strategy was undertaken with an experienced information scientist. What was uploaded was a formatting error in the supplementary files, which has since been amended. Thank you.

Reviewer: 2
Dr. J. Fluss, Univ Hosp Geneva

Comments to the Author:

I have accepted to review this manuscript having explored both the topic of literacy and SES, as well as the field of CP. The manuscript is very well written and brings very important message for any professionals involve in the care of CP children in the setting of family living in or coming from LMICs.

It would have been maybe meaningful for the authors (and for the readership) to have a better explanation of the Ophelia framework, and eventually to discuss it a bit more within the introduction.
Our response: Thank you, we have amended the manuscript to provide a more detailed explanation of the framework. Please see pages 3 and 4.

As many potential readers are presumably coming from high income countries, and encounter in their practice more and more immigrant children with CP from LMICs, it could be of interest to discuss briefly this issue (has it been studied?).

Our response: Thank you for this thought-provoking comment. Given how heavily health literacy relies on the external context, immigrant parents of children with CP face their own unique challenges (such as navigating an unfamiliar health service, communicating in a second language, diminished social support,

and unfamiliar cultural context in healthcare settings). There is also the matter of immigrant parents having economic means to emigrate (meaning they are likely of increased SES in their home country). Due to the variation in the presentation of this group versus those who still reside in LMICs, a comment on this important issue may not receive the justice it deserves within this paper.

practical points:
the authors should clarify why they do not cover all domains in the result section (unclear).
Our response: Thank you for this helpful comment. Please see page 7 (results section) where we have addressed this following review.
